# Featured intestinal microbiota associated with hepatocellular carcinoma in various liver disease states

**DOI:** 10.3389/fimmu.2025.1674838

**Published:** 2025-10-06

**Authors:** Xiu Sun, Zhewen Zhou, Xin Chi, Danying Cheng, Yuanyuan Zhang, Yifan Xu, Yanxu Hao, Ying Duan, Wei Li, Yingying Zhao, Shunai Liu, Ming Han, Xi Wang, Song Yang, Calvin Q. Pan, Huichun Xing

**Affiliations:** ^1^ Center of Liver Diseases Division 3, Beijing Ditan Hospital, Capital Medical University, Beijing, China; ^2^ Shanxi Bethune Hospital, Shanxi Academy of Medical Sciences, Third Hospital of Shanxi Medical University, Tongji Shanxi Hospital, Taiyuan, China; ^3^ National Center for Infectious Diseases, Beijing, China; ^4^ Beijing Key Laboratory of Emerging Infectious Diseases, Institute of Infectious Disease, Beijing Ditan Hospital, Capital Medical University, Beijing, China; ^5^ Division of Gastroenterology and Hepatology, New York University Langone Health, New York University School of Medicine, New York, NY, United States; ^6^ Peking University Ditan Teaching Hospital, Beijing, China

**Keywords:** intestinal microbiota, 16S rDNA, chronic hepatitis B, hepatocellular carcinoma, liver cirrhosis

## Abstract

**Objective:**

This study aimed to identify distinct intestinal microbiota associated with hepatocellular carcinoma (HCC) and to construct a predictive model for HCC.

**Methods:**

A case-control study was conducted including patients with chronic hepatitis B (CHB), liver cirrhosis (LC), HCC, and healthy controls (HC). Fecal 16S rDNA sequences were analyzed using bioinformatics approaches. Specific intestinal microbiota were identified through stratified analysis, and a predictive model was subsequently constructed.

**Results:**

A total of 152 subjects were enrolled, including CHB (n = 33), LC (n = 59; 25 compensated cirrhosis, CC; 34 decompensated cirrhosis, DC), HCC (n = 30; 5 CHB-HCC, 9 CC-HCC, and 16 DC-HCC), and HC (n = 30). A significant overall difference in alpha diversity was observed across the groups (Chao1: P = 0.010,ϵ²= 0.056; ACE: P = 0.016,ϵ²= 0.049). In the CHB-HCC, CC-HCC, and DC-HCC groups, the abundance of *Bacteroides*, *Prevotella*, and *Faecalibacterium* gradually decreased, whereas *Klebsiella*, *Haemophilus*, and *Streptococcus* increased. Comparison of CHB vs. CHB-HCC, CC vs. CC-HCC, and DC vs. DC-HCC revealed consistent microbial shifts across disease stages. In particular, *Roseburia*, *Veillonella*, *Megasphaera*, and *Paraprevotella* were increased irrespective of liver disease stage. By combining microbiota profiles with clinical indicators, we developed a predictive nomogram that achieved an AUC of 0.865 in the training cohort and 0.848 in the external validation cohort.

**Conclusion:**

Intestinal microbiota were associated not only with liver disease stage but also with the occurrence of HCC itself. Characteristic microbiota may serve as effective biomarkers for predicting HCC.

## Graphical Abstract

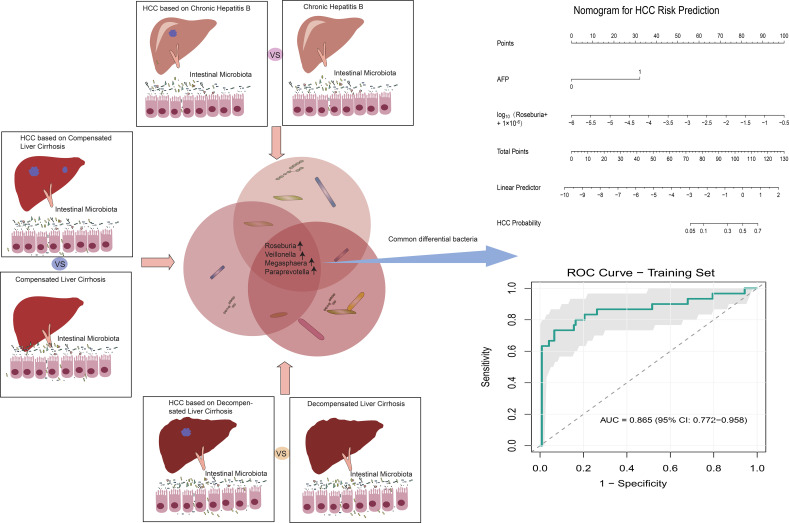

## Highlights

- The intestinal microbiota associated with hepatitis B-related HCC may reflect not only the development of HCC itself but also the underlying stage of liver disease.
- Characteristic microbiota associated with HCC, independent of liver disease stage, were identified through stratified comparisons.
- A predictive model integrating characteristic microbiota and clinical indicators accurately assessed the risk of HCC.

## 1 Introduction

Primary liver cancer is the sixth most common cancer worldwide, accounting for 4.7% of all cancer cases. It is also the third leading cause of cancer-related death, responsible for approximately 906,000 new cases and 830,000 deaths annually (1). Hepatocellular carcinoma (HCC), the predominant histological subtype of primary liver cancer, accounts for 75–85% of cases. Globally, about 850,000 new HCC cases are diagnosed each year, and the estimated 5-year survival rate is only 20% (1, 2). HCC is a progressive and aggressive malignancy. Although treatment strategies such as surgery, chemoradiotherapy, immunotherapy, and targeted therapy have advanced in recent years, the prognosis of patients with HCC remains poor due to high rates of recurrence and metastasis. In particular, systemic therapies have advanced from tyrosine kinase inhibitors (TKIs), such as sorafenib and lenvatinib, which provided survival benefit but were constrained by resistance, to immune checkpoint inhibitors (ICIs), which achieved durable responses in a subset of patients. Landmark clinical trials further established combination regimens as new standards of care. For example, atezolizumab plus bevacizumab (IMbrave150) and tremelimumab plus durvalumab (HIMALAYA) significantly improved overall survival compared with sorafenib. Despite these advances, many patients continue to experience poor outcomes, underscoring the urgent need for deeper mechanistic studies and innovative therapeutic approaches (3). Therefore, elucidating the molecular mechanisms underlying HCC initiation and progression and developing effective diagnostic and therapeutic strategies remain essential.

The development of HCC is driven by multiple factors, including viral infections, metabolic syndrome, alcoholic liver disease, and non-alcoholic fatty liver disease. Among these, chronic hepatitis B virus (HBV) infection remains the leading cause of HCC in China (1). Even with effective viral suppression through antiviral therapy, some patients with HBV-related HCC still experience tumor progression, indicating that additional mechanisms contribute to hepatocarcinogenesis. Recent evidence highlights the gut microbiota as a key regulator of immunity, inflammation, and metabolism, contributing to HCC initiation and progression (4–6). Consequently, gut microbial alterations have become an emerging research focus. However, whether these microbial changes follow consistent patterns across different liver disease stages remains unclear.

Accumulating studies, including animal models, have shown that intestinal microbiota influence the occurrence and development of HCC through bidirectional gut–liver interactions (7). Human studies also reported significant microbial alterations in patients with HCC (8, 9). Notably, microbial community structures vary across liver disease stages; for instance, distinct differences have been observed between chronic hepatitis B (CHB) and liver cirrhosis (LC) (10, 11). HCC can develop in diverse disease backgrounds, including CHB, compensated cirrhosis (CC), and decompensated cirrhosis (DC). Nevertheless, most previous studies did not evaluate how underlying liver disease status affects intestinal microbiota in HCC, which may introduce ambiguity. Indeed, microbial profiles in patients with HCC are influenced not only by the tumor itself but also by the underlying liver disease stage.

Therefore, stratified analysis is necessary to disentangle these effects. In this study, we compared microbial compositions between CHB and CHB-HCC, CC and CC-HCC, and DC and DC-HCC to identify characteristic intestinal microbiota associated with HCC independent of liver disease stage. Such stratification enables the exclusion of confounding effects from different liver disease backgrounds, thereby facilitating the identification of HCC-specific microbial signatures. This approach may deepen our understanding of the microbiota–HCC relationship and provide a basis for novel diagnostic and therapeutic strategies targeting intestinal microbiota in HCC.

## 2 Materials and methods

### 2.1 Study participants

This single-center case-control study was approved by the Medical Ethics Committee of Beijing Ditan Hospital, Capital Medical University (DT-IRB-2018-04001). Written informed consent was obtained from all participants prior to enrollment. The study was conducted in accordance with Good Clinical Practice (GCP) guidelines.

Patients who attended outpatient or inpatient services at Beijing Ditan Hospital, Capital Medical University, between July 2019 and August 2021 were enrolled. Sample size estimation was based on relevant literature, with details provided in the **Supplementary Methods**. Based on literature-reported cirrhosis dysbiosis ratio (CDR) means in healthy controls (2.05), compensated cirrhosis (0.89), decompensated cirrhosis (0.66), and inpatients (0.32), with a common SD of 1.5, an *a priori* one-way ANOVA power analysis (α = 0.05, power = 0.90) indicated N ≈ 100 (≈25 per group). We enrolled 257 participants, which were allocated as 60% training (n = 152) and 40% validation (n = 105) cohorts across HC, CHB, LC, and HCC groups. All participants were over 18 years of age. All patients in this study received standardized treatments recommended by clinical guidelines, including antiviral with entecavir (ETV), hepatoprotective, and supportive therapies. CHB was defined as hepatitis B surface antigen or HBV DNA positivity for more than 6 months, accompanied by persistent or recurrent abnormalities in alanine aminotransferase (ALT) or evidence of significant hepatic inflammation and necrosis on histology, or fibrosis suggested by histology or non-invasive markers. Patients who met the criteria for CHB and had radiological or pathological evidence of HCC, without prior interventional, surgical, or systemic antitumor therapy, were classified into the HCC group. Patients without HCC but with radiological evidence of cirrhosis, portal hypertension, or splenomegaly, with or without ascites, and without bacterial infection, hepatic encephalopathy, or gastrointestinal bleeding, were classified into the LC group. Healthy controls had normal physical examination, liver and kidney function, and no history of heart, brain, kidney, or lung disease. Exclusion criteria were as follows: (a) alcoholic liver disease, autoimmune liver disease, fatty liver disease, chronic gastrointestinal disease, or other viral infections such as hepatitis C virus (HCV) or human immunodeficiency virus (HIV); (b) diabetes, obesity, or metabolic syndrome; (c) use of antibiotics, probiotics, or proton pump inhibitors within one month; and (d) pregnancy or lactation.

### 2.2 Study design

The study consisted of four parts. First, patients with HCC were enrolled as the study group, while HC, CHB, and LC patients served as controls to investigate microbiota alterations associated with HCC. Second, microbial profiles of CHB-HCC, CC-HCC, and DC-HCC were compared to examine microbiota differences in HCC under different liver disease backgrounds. Third, microbial profiles were compared between patients with and without HCC within the same liver disease stage (CHB-HCC vs. CHB, CC-HCC vs. CC, DC-HCC vs. DC) to identify HCC-associated microbiota independent of liver disease stage. Finally, a predictive model for HCC was developed by integrating clinical indicators and microbiota features, and its clinical utility was validated.

### 2.3 Data collection and specimen collection

All participants who met the inclusion criteria were evaluated at their first visit for demographic characteristics, medical history, and physical examination. Participants were instructed to record their dietary patterns for one month prior to the visit. Fresh fecal specimens were collected in sterile containers and transported under cold-chain conditions to the specimen biobank. All samples were stored at –80 °C within 30 minutes of collection. Fasting peripheral venous blood samples were collected concurrently with stool specimens.

### 2.4 Detection method

Liver function, renal function, HBV serum markers, and complete blood counts were assessed on the day of collection by the Laboratory Department of Beijing Ditan Hospital, Capital Medical University. Complete blood counts were measured using an automatic hematology analyzer (Sysmex XN-10). Liver and renal function tests were performed with an automatic biochemical analyzer (Hitachi 7600-020). HBV serum markers and tumor markers were analyzed using an automatic chemiluminescence analyzer (Beckman Coulter ACCESS2). Coagulation parameters were assessed with an automatic blood coagulation analyzer (Werfen ACLTOP750CTS). HBV DNA load was quantified using a gene amplification system with supporting reagents (Roche Cobas).

Microbial DNA was extracted from fecal samples, and the V3–V4 region of the 16S rRNA gene was amplified, purified, and used to construct DNA libraries, which were subsequently sequenced on an Illumina NovaSeq platform. Further details are provided in the Supplementary Methods.

### 2.5 Data analysis and statistical consideration

Clean data were generated after quality trimming and filtering of raw sequencing reads. Sequences were clustered into operational taxonomic units (OTUs) at 97% similarity, and taxonomic annotation was performed based on OTU sequences. Diversity indices were calculated using QIIME software (version 1.9.1). Microbial differences were analyzed using the linear discriminant analysis (LDA) effect size (LEfSe) method, with an LDA score (log_10_) ≥ 2 as the cutoff.

All statistical analyses were performed using R software (version 3.6.3). Categorical variables were analyzed using chi-square tests, while continuous variables were compared using Kruskal–Wallis tests. Variables that were significant in univariate logistic regression (FDR q < 0.05) were included in the multivariate analysis. To address potential complete separation issues, Firth’s penalized-likelihood logistic regression was employed. The abundance of *Roseburia* was log_10_(x + 1×10^-6^) transformed to account for zero values. The HCC predictive model (PM-HCC) was developed and presented as a nomogram. Model performance was evaluated using ROC curve analysis and calibration plots. A p-value of < 0.05 was considered statistically significant.

## 3 Results

### 3.1 Clinical characteristics of the patients

**Figure 1:**
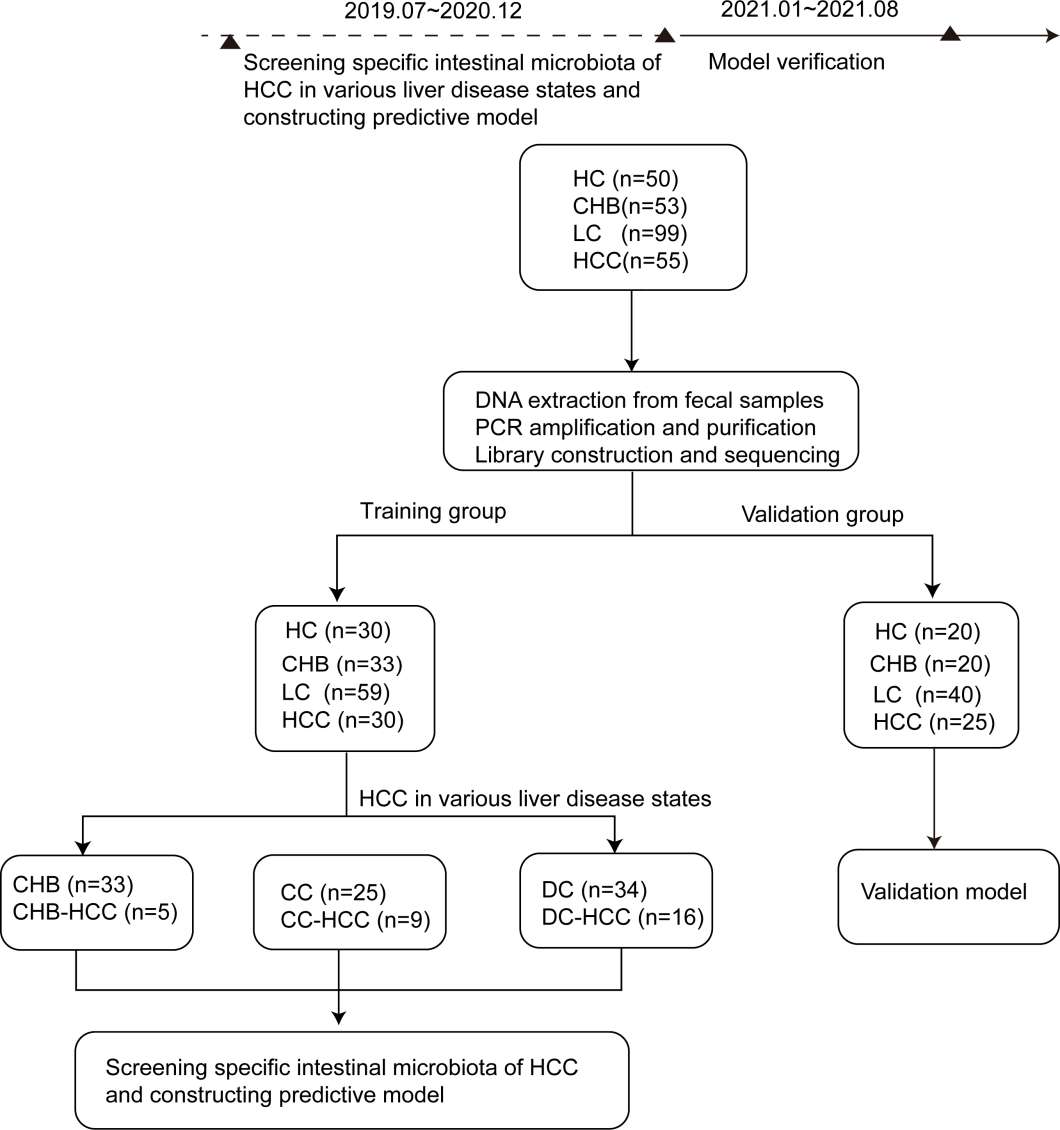
Research design and flow chart. According to the strict inclusion and exclusion criteria, 257 stool samples were included, including 50 cases of HC, 53 cases of CHB, 99 cases of LC and 55 cases of HCC. According to the time order of inclusion, they are divided into training group (n=152, including 30 cases of healthy controls, 33 cases of CHB, 59 cases of LC, 30 cases of HCC) and verification group (n=105, including 20 cases of healthy controls, 20 cases of CHB, 40 cases of LC and 25 cases of HCC).The differences of intestinal microbiota among the HC,CHB,LC and HCC groups in training group were compared. Of the 30 patients with HCC in training group, 5 cases were CHB-HCC, 9 cases were CC-HCC and 16 cases were DC-HCC. The intestinal microbiota of patients with or without HCC under different liver disease states were compared. Finally, the specific microbiota of HCC were screened, and the prediction model was constructed and verified in the verification group. CHB, chronic hepatitis B; HCC, hepatocellular carcinoma; CHB-HCC, HCC based on chronic hepatitis B; CC, compensatory cirrhosis; CC-HCC, HCC based on compensated liver cirrhosis; DC, decompensatory cirrhosis; DC-HCC, HCC based on decompensated liver cirrhosis; HC, healthy control; LC, liver cirrhosis.

A total of 257 participants were enrolled, comprising 55 HCC, 53 CHB, 99 LC, and 50 HC. Of these, 30 HCC, 33 CHB, 59 LC, and 30 HC admitted before January 1, 2021, were assigned to the training cohort for model development, whereas 25 HCC, 20 CHB, 40 LC, and 20 HC admitted thereafter were allocated to the validation cohort. The study design is illustrated in **Figure 1**. Demographic characteristics, dietary patterns, and clinical data of the participants are summarized in **Table 1**, and details on tumor size and number for the 30 patients with HCC are provided in **Supplementary Table S1**. In the training cohort, aspartate aminotransferase (AST) levels were higher, while albumin (ALB) levels, red blood cell (RBC) and white blood cell (WBC) counts, hemoglobin (HGB) levels, and platelet (PLT) counts were lower in the LC and HCC groups compared with the HC and CHB groups (P < 0.05). Additionally, HBV DNA load was higher in the LC and HCC groups than in the CHB group (P < 0.05). These findings indicated more advanced liver dysfunction in the LC and HCC groups.

**Table 1:** Baseline clinical characteristics of the patients.

Characteristics	HC (n=30)	CHB (n=33)	LC (n=59)	HCC (n=30)
Gender(F/M)	12/18	10/23	21/38	6/24
Age	48.1 ± 9.0	47.4 ± 8.2	50.9 ± 9.1	52.8 ± 8.6
BMI`(x̄ ± S)	23.37 ± 2.9	24.1 ± 1.8	24.3 ± 1.7	24.6 ± 1.7
ALT(U/L)	22.7 ± 10.6	23.3 ± 9.4	40.1 ± 60.8	32.3 ± 24.0
AST(U/L)	19.4 ± 5.0^ab^	21.5 ± 6.7^cd^	47.1 ± 59.5^ac^	45.3 ± 30.1^bd^
ALB(g/L)	45.3 ± 2.8^ab^	46.8 ± 3.7^cd^	36.7 ± 7.8^ac^	37.3 ± 7.3^bd^
TBil(µmol/L)	14.4 ± 4.6^a^	14.7 ± 5.0^b^	32.1 ± 31.2^ab^	18.8 ± 7.5
Cr(µmol/L)	66.4 ± 13.0	71.2 ± 11.6	67.5 ± 14.1	71.3 ± 10.9
HbeAg	0^abc^	6.6 ± 13.7^a^	76.3 ± 230.6^b^	7.3 ± 31.5^c^
WBC(10^9^/L)	5.2 ± 0.9^ab^	5.7 ± 1.0^bc^	3.8 ± 1.8^a^	4.4 ± 2.0^c^
RBC(10^12^/L)	4.8 ± 0.3^ab^	5.0 ± 0.4^cd^	4.1 ± 0.8^ac^	4.2 ± 0.7^bd^
HGB(g/L)	145.6 ± 11.4^ab^	152.4 ± 18.7^cd^	126.2 ± 22.75^ac^	130.1 ± 19.4^bd^
PLT(10^9^/L)	190.6 ± 40.8^ab^	209.4 ± 57.0^cd^	81.6 ± 30.8^ac^	124.1 ± 77.5^bd^
AFP(ng/ml)				
≤8.78	30(100%)^ab^	33(100%)^cd^	41(69.5%)^ace^	7(23.3%)^bde^
>8.78	0(0%)	0(0%)	18(30.5%)	23(76.7%)
Log10HBV DNA(IU/ml)	–	1.09 ± 0.09^ab^	2.7 ± 1.86^a^	2.21 ± 1.51^b^
Dietary habit	Mix	Mix	Mix	Mix

Continuous variables were expressed as means ± standard deviation. Superscript letters indicated a significant difference (P < 0.05). BMI, body mass index; WBC, white blood cell; RBC, red blood cell; HGB, hemoglobin; PLT, blood platelet; ALB, albumin; TBil, total bilirubin; ALT, alanine aminotransferase; AST, aspartate aminotransferase; AFP, alpha fetoprotein; HBeAg, HBV e antigen; Cr, creatinine; HC, healthy control; CHB, chronic hepatitis B; LC, liver cirrhosis; HCC, hepatocellular carcinoma.

### 3.2 The features of intestinal microbiota in patients with CHB, LC, and HCC

**Figure 2:**
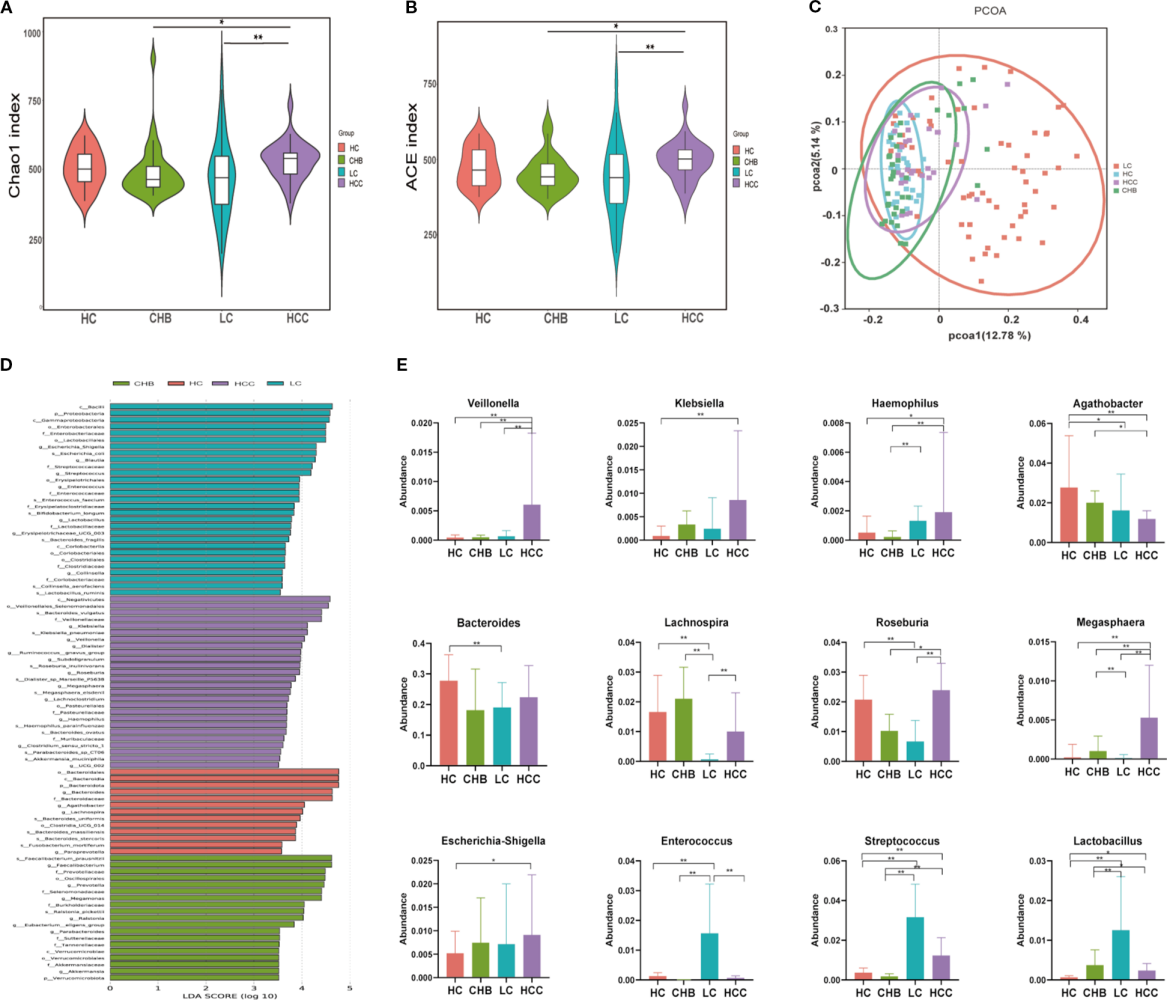
Analysis of intestinal microbiota diversity and difference in the healthy group, chronic hepatitis B, liver cirrhosis, and HCC. **(A)** Chao1 index; **(B)** ACE index; **(C)** Beta diversity was calculated using unweighted uniFrac by PCoA; **(D)** Differential taxa identified by LEfSe analysis (LDA>3.5); **(E)** The changes of bacteria in the different groups. HC, healthy control; CHB, chronic hepatitis B; LC, liver cirrhosis; HCC, hepatocellular carcinoma. * p<0.05; ** p<0.01.

The intestinal microbiota profiles of the CHB, LC, and HCC groups are summarized as follows. Rarefaction and rank abundance curves indicated that sequencing depth was sufficient and species distribution was uniform across samples (**Supplementary Figures S1A, B**). A significant overall difference in alpha diversity was observed across the groups (Chao1: P = 0.010, ϵ²= 0.056; ACE: P = 0.016, ϵ²= 0.049). Despite the small effect sizes, a striking and consistent pattern was revealed: the indices were significantly higher in HCC than in both CHB (Chao1: P = 0.012; ACE: P = 0.012) and LC (Chao1: P = 0.0015; ACE: P = 0.0024) (**Figures 2A, B**), suggesting a distinct alteration in the gut microbiome associated with HCC. Beta diversity was assessed using unweighted UniFrac distance-based principal coordinate analysis (PCoA) to visualize microbial community dissimilarities among samples. The HC group showed tight clustering, while the CHB group exhibited a composition similar to HC, and the LC group showed greater dissimilarity. In contrast, HCC samples did not show increased dissimilarity; instead, their profiles were comparable to those of HC and CHB, suggesting specific microbial alterations in HCC (**Figure 2C**). To statistically validate the observed structural differences, a PERMANOVA analysis based on unweighted UniFrac distances was conducted. The results showed that the “Group” factor was a statistically significant predictor of microbial community composition (R² = 0.108, F = 5.968, P = 0.001), indicating substantial overall differences in the gut microbiota across disease stages.

Relative abundances of intestinal microbiota in HC, CHB, LC, and HCC are shown in **Supplementary Figures S2A, B**. Across all groups, *Firmicutes*, *Bacteroidota*, *Proteobacteria*, and *Actinobacteria* were the dominant phyla (>90% of total abundance), while *Bacteroides*, *Faecalibacterium*, and *Prevotella* were common dominant genera (>30% of abundance). Overall comparisons revealed distinct group-specific features: HC was enriched in *Bacteroides*, *Lachnospira*, and *Agathobacter*; CHB in *Faecalibacterium*, *Prevotella*, and *Megamonas*; LC in *Escherichia-shigella*, *Streptococcus*, and *Enterococcus*; and HCC in *Klebsiella*, *Veillonella*, *Roseburia*, and *Megasphaera* (**Figure 2D**). These results indicate clear compositional shifts in gut microbiota across different disease states. **Figure 2E** illustrates bacterial differences among the groups. Compared with the other groups, the abundances of *Veillonella*, *Klebsiella*, and *Haemophilus* were significantly higher in the HCC group, whereas *Agathobacter* exhibited the lowest abundance. The abundance of *Bacteroides* was lower in the LC group than in the HC group, whereas *Escherichia-shigella* was higher in the HCC group. The abundances of *Lachnospira*, *Roseburia*, and *Megasphaera* were significantly lower in the LC group than in the CHB and HCC groups. Conversely, the abundances of *Streptococcus*, *Enterococcus*, and *Lactobacillus* were significantly higher in the LC group than in the other groups. These findings suggest distinct alterations in the intestinal microbiota associated with HBV-related HCC.

### 3.3 The features of intestinal microbiota in patients with HCC in different liver disease states

The above findings indicated variations in the intestinal microbiota among individuals with different liver disease states, including CHB, LC and HCC. HCC can arise from various underlying liver diseases, including CHB, CC and DC. Therefore, it was necessary to determine whether alterations in the intestinal microbiota in HCC were attributable to underlying liver disease states or to the presence of HCC itself. To address this, we evaluated the intestinal microbiota profiles of HCC across different liver disease states.

**Figure 3:**
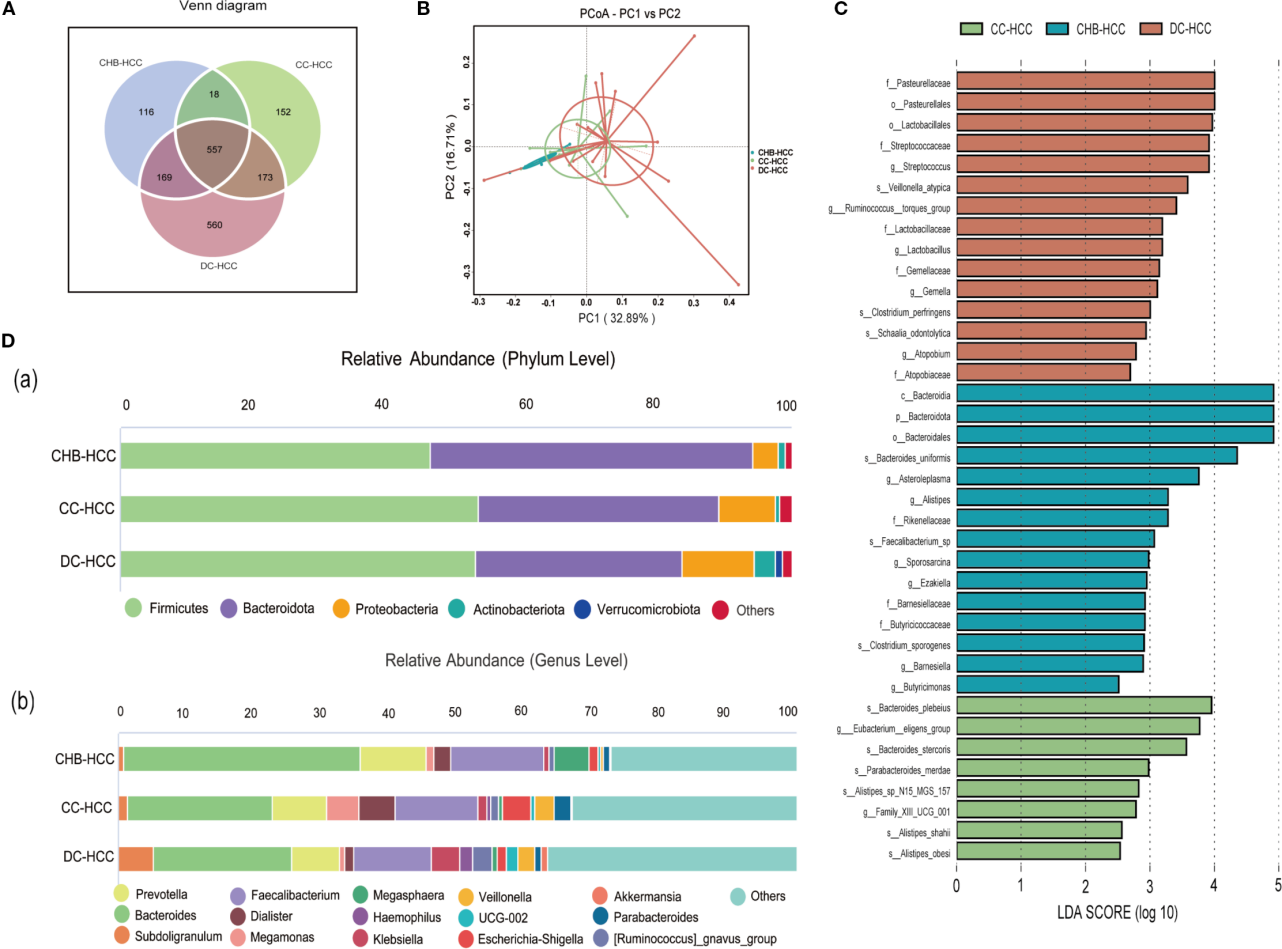
Analysis of intestinal microbiota diversity, composition, and difference in three different states of HCC. **(A)**Venn diagram; **(B)** Beta diversity was calculated using weighted UniFrac by PCoA; **(C)** Differential taxa identified by LEfSe analysis (LDA>2.5); **(D)** Relative abundance histogram (a. phylum level and **(B)** genus level). CHB-HCC, hepatocellular carcinoma based on chronic hepatitis B; CC-HCC, hepatocellular carcinoma based on compensated liver cirrhosis; DC-HCC, hepatocellular carcinoma based on decompensated liver cirrhosis.

According to the Venn diagram, 557 operational taxonomic units (OTUs) were shared among the CHB-HCC, CC-HCC, and DC-HCC groups, while 116 OTUs were unique to the CHB-HCC group, 152 OTUs were unique to the CC-HCC group, and 560 OTUs were unique to the DC-HCC group (**Figure 3A**). No significant differences were observed in α diversity among patients with HCC across the three liver disease states (P > 0.05), PCoA based on weighted UniFrac distance revealed partially overlapping clustering patterns among the CHB-HCC, CC-HCC, and DC-HCC groups (**Figure 3B**). PERMANOVA analysis using unweighted UniFrac distance confirmed the absence of statistically significant separation at the whole-community level (F = 1.142, R² = 0.081, P = 0.178), which was consistent with the limited segregation observed in the PCoA plot. Despite this overall structural similarity, we considered that the three HCC subtypes (CHB-HCC, CC-HCC, and DC-HCC), which represent a progressive pathological spectrum from chronic hepatitis through compensated cirrhosis to decompensated cirrhosis, might still harbor specific microbial taxa associated with disease progression. To explore this possibility, we subsequently performed LEfSe analysis. The LEfSe analysis showed that the CHB-HCC group was enriched in *Asteroleplasma* (LDA = 3.82, P = 0.02), *Alistipes* (LDA = 3.26, P = 0.004), and *Sporosarcina* (LDA = 3.03, P = 0.049); the CC-HCC group was enriched in *Eubacterium eligens* (LDA = 4.16, P = 0.009); and the DC-HCC group was enriched in *Streptococcus* (LDA = 3.97, P = 0.006) and *Lactobacillus* (LDA = 3.57, P = 0.03) at the genus level (**Figure 3C**). Relative abundances of microbiota at the phylum and genus levels in HCC across the three liver disease states are illustrated in **Figure 3D**. In the CHB-HCC, CC-HCC, and DC-HCC groups, the relative abundances of *Bacteroidota* were 48.1%, 35.8%, and 30.9%, respectively; *Proteobacteria* accounted for 3.7%, 8.4%, and 10.9%, respectively; and *Veillonella* accounted for 0.27%, 2.88%, and 2.53%, respectively. Overall, the abundances of potentially pathogenic bacteria such as *Klebsiella*, *Haemophilus*, and *Streptococcus* were highest in the DC-HCC group, whereas *Bacteroides*, *Prevotella*, and *Faecalibacterium* were lowest. These findings suggest that the unique composition of intestinal microbiota was associated not only with HCC but also with the underlying liver disease state.

### 3.4 Identification of specific microbiological markers in patients with HCC itself

Different liver disease states can influence the composition of the intestinal microbiota. To identify microbiological markers specific to HCC itself, additional stratified analyses were performed. We hypothesized that within the same liver disease state, alterations in the intestinal microbiota of patients with HCC might be more closely related to the presence of HCC itself. Therefore, we compared intestinal microbiota within the same liver disease state, specifically CHB-HCC versus CHB, CC-HCC versus CC, and DC-HCC versus DC.

#### 3.4.1 Specific microbiological markers associated with CHB-HCC

Clinical and demographic characteristics of patients with CHB and CHB-HCC are shown in **Supplementary Table S2**. No significant differences were observed in age, sex, or BMI between the two groups. ALB levels and RBC counts were lower in CHB-HCC than in CHB, whereas INR values were higher (P = 0.029, 0.040, and 0.005, respectively). AFP levels were significantly higher in CHB-HCC than in CHB.

**Figure 4:**
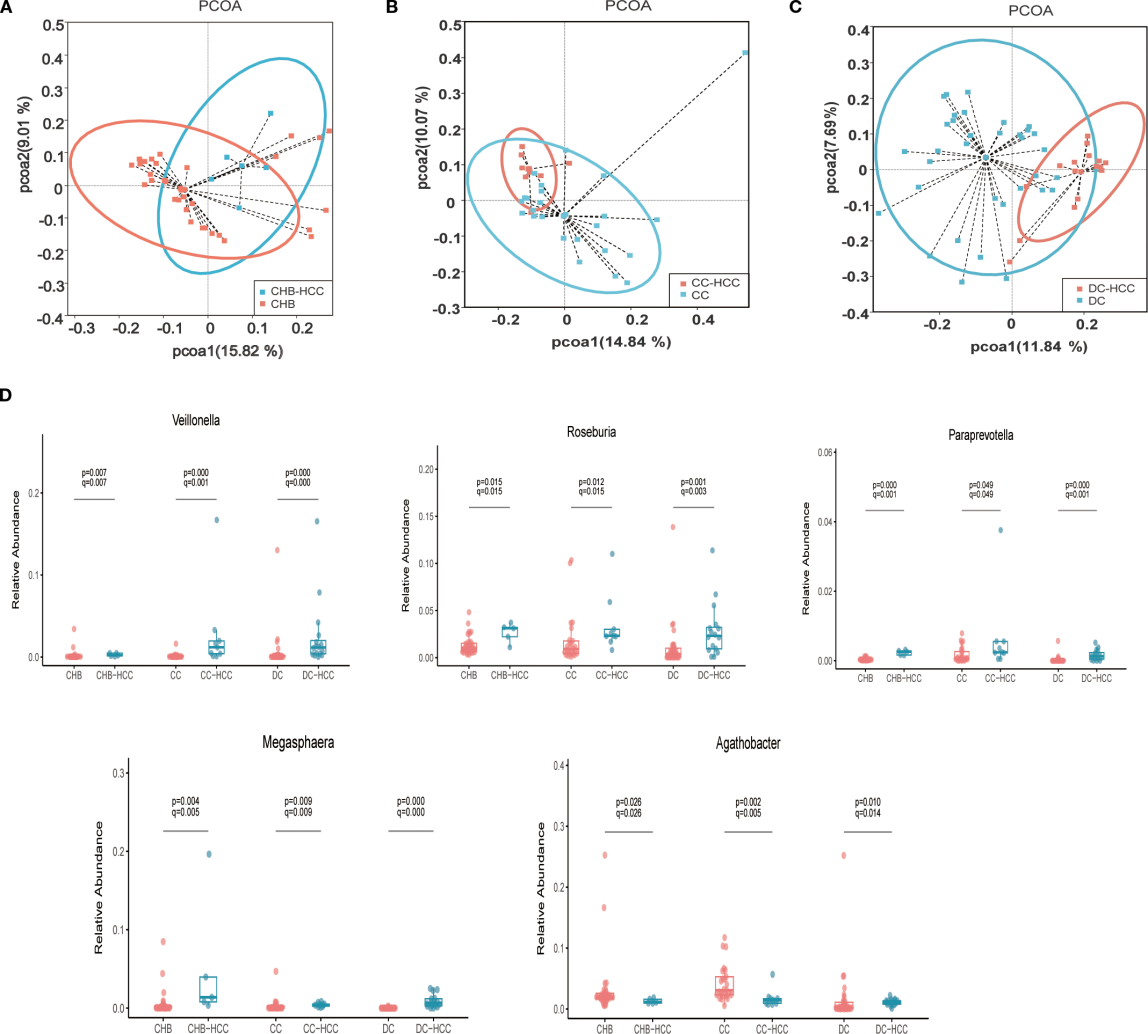
Comparison of intestinal microbiota profiles in different liver disease states with and without HCC. **(A)** Principal coordinate Analysis of unweighted unifrac distance between CHB and CHB-HCC; **(B)** Principal coordinate Analysis of unweighted unifrac distance between CC and CC-HCC; **(C)** Principal coordinate Analysis of unweighted unifrac distance between DC and DC-HCC; **(D)** Relative abundance box diagram of bacteria. The abundance of *Roseburia*, *Veillon*ella, *Megasphaera* and *Paraprevotella* were higher in three states of HCC (CHB-HCC, CC-HCC, DC-HCC) than that in NHCC (CHB, CC, DC) respectively. CHB, chronic hepatitis B; CC, chronic hepatitis B; DC, decompensated cirrhosis; HCC, hepatocellular carcinoma; CHB-HCC, HCC based on chronic hepatitis B; CC-HCC, HCC based on compensated liver cirrhosis; DC-HCC, HCC based on decompensated liver cirrhosis; NHCC, non-HCC (CHB, CC, DC).

Relative abundances of intestinal microbiota at the phylum and genus levels in patients with CHB and CHB-HCC are shown in **Supplementary Figures S3A, B**. PCoA based on unweighted UniFrac distance showed distinct clustering of the intestinal microbiota between the CHB and CHB-HCC groups (**Figure 4A**). This visual separation was further supported by PERMANOVA based on Bray–Curtis dissimilarity, which confirmed a statistically significant difference in microbial community structure between the two groups (F = 1.69, R² = 0.045, P = 0.046). LEfSe analysis revealed that *Agathobacter* (LDA = 3.93, P = 0.026) and *Eubacterium ruminantium* group (LDA = 3.14, P = 0.04) were enriched in the CHB group. In contrast, the CHB-HCC group was enriched in more taxa, including *Megasphaera* (LDA = 4.38, P = 0.0035), *Asteroleplasma* (LDA = 4.00, P = 0.012), *Roseburia* (LDA = 3.81, P = 0.017), *Paraprevotella* (LDA = 3.01, P = 0.0003), and *Veillonella* (LDA = 2.96, P = 0.007) (**Supplementary Figure S4**). These findings suggest that in CHB, the intestinal microbiota became more complex after the development of HCC, accompanied by alterations in specific bacterial taxa.

#### 3.4.2 Specific microbiological markers associated with CC-HCC

No significant differences were observed in age, sex, or BMI between the CC and CC-HCC groups. Similarly, no significant differences were observed in ALT, AST, TBil, ALB, or PLT counts between the two groups (P > 0.05). AFP levels were significantly higher in the CC-HCC group than in the CC group (P < 0.05, **Supplementary Table S3**).

Relative abundances of intestinal microbiota at the phylum and genus levels in patients with CC and CC-HCC are shown in **Supplementary Figures S5A, B**. PCoA based on unweighted UniFrac distance demonstrated distinct microbial community structures between the CC and CC-HCC groups (**Figure 4B**). PERMANOVA further confirmed this distinct separation, revealing a statistically significant difference between the two groups (F = 2.3333, R² = 0.07, P = 0.001). This divergence suggests that the intestinal microbial community structure differs substantially between the two groups. LEfSe analysis revealed that the CC group was enriched in *Agathobacter* (LDA = 4.12, P = 0.0017), *Blautia* (LDA = 4.07, P = 0.0007), *Lactobacillus* (LDA = 4.06, P = 0.0026), *Ralstonia* (LDA = 3.98, P = 0.009), *Bifidobacterium* (LDA = 3.97, P = 0.0002), and *Dorea* (LDA = 3.45, P = 0.0003). In contrast, the CC-HCC group was enriched in *Veillonella* (LDA = 4.14, P = 0.00035), *Roseburia* (LDA = 3.93, P = 0.012), *Lachnoclostridium* (LDA = 3.79, P = 0.0008), *Klebsiella* (LDA = 3.70, P = 0.037), *Lachnospira* (LDA = 3.53, P = 0.044), *Paraprevotella* (LDA = 3.38, P = 0.04), and *Megasphaera* (LDA = 3.17, P = 0.01) (**Supplementary Figure S6**). These results indicate that the intestinal microbiota underwent specific alterations in response to the development of HCC within the context of CC.

#### 3.4.3 Specific microbiological markers associated with DC-HCC

No significant differences were observed in age, sex, or BMI between the DC and DC-HCC groups. Total bilirubin levels were higher in the DC group than in the DC-HCC group (P = 0.046), whereas no significant differences were observed in ALT, AST, ALB, or PLT counts (P > 0.05). AFP levels were significantly higher in the DC-HCC group than in the DC group (P < 0.05, **Supplementary Table S4**).

Relative abundances of intestinal microbiota at the phylum and genus levels in patients with DC and DC-HCC are shown in **Supplementary Figures S7A, B**. Alpha diversity (Chao1 index) of intestinal microbiota was higher in the DC-HCC group than in the DC group (P < 0.05, **Supplementary Figure S8**). PCoA based on unweighted UniFrac distance revealed distinct microbial community structures between the DC and DC-HCC groups (**Figure 4C**). PERMANOVA further confirmed this observation, revealing a statistically significant difference between the two groups (F = 4.3303, R² = 0.083, P = 0.001). LEfSe analysis showed that the DC group was enriched in *Bifidobacterium* (LDA = 4.37, P = 0.012), *Blautia* (LDA = 4.29, P = 0.02), *Enterococcus* (LDA = 4.10, P = 3.90 × 10^-5^), *Agathobacter* (LDA = 3.60, P = 0.01), *TM7x* (LDA = 3.48, P = 0.03), and *Alistipes* (LDA = 3.47, P = 0.02). In contrast, the DC-HCC group was enriched in *Prevotella* (LDA = 4.45, P = 1.92 × 10^-5^), *Faecalibacterium* (LDA = 4.42, P = 0.004), *Ruminococcus gnavus* group (LDA = 4.08, P = 0.001), *Roseburia* (LDA = 3.97, P = 0.0013), *Veillonella* (LDA = 3.90, P = 3.82 × 10^-5^), *Lachnospira* (LDA = 3.88, P = 1.30 × 10^-5^), *Lachnoclostridium* (LDA = 3.83, P = 0.0001), *Megasphaera* (LDA = 3.65, P = 1.39 × 10^-6^), *Dialister* (LDA = 3.58, P = 2.87 × 10^-6^), and *Paraprevotella* (LDA = 2.81, P = 0.006) (**Supplementary Figure S9**). Similarly, the intestinal microbiota underwent specific alterations following the development of HCC within the context of DC.

The stratified analysis (based on Lefse analysis) revealed significant alterations in the intestinal microbiota of individuals with HCC, irrespective of the underlying liver disease state. Notably, certain bacteria such as *Roseburia*, *Veillonella*, *Megasphaera*, and *Paraprevotella* were consistently enriched in all CHB-HCC, CC-HCC, and DC-HCC groups, whereas *Agathobacter* was relatively reduced. We performed Wilcoxon tests to further analyze the aforementioned bacterial genera, followed by false discovery rate (FDR) correction. The results demonstrated that, compared with the non-HCC group, *Roseburia*, *Veillonella*, *Megasphaera*, and *Paraprevotella* were significantly enriched in the HCC group (all q < 0.05) (**Figure 4D**), suggesting that these four bacterial genera may be specifically associated with HCC and independent of the underlying liver disease status. Notably, unlike the declining trends observed in CHB vs. CHB-HCC and CC vs. CC-HCC, the relative abundance of *Agathobacter* was significantly higher in DC-HCC patients than in those with DC, indicating that *Agathobacter* is not a bacterial feature consistently associated with HCC across the three distinct disease states (**Figure 4D**).

### 3.5 Efficiency of microbiota in predicting HCC

**Figure 5:**
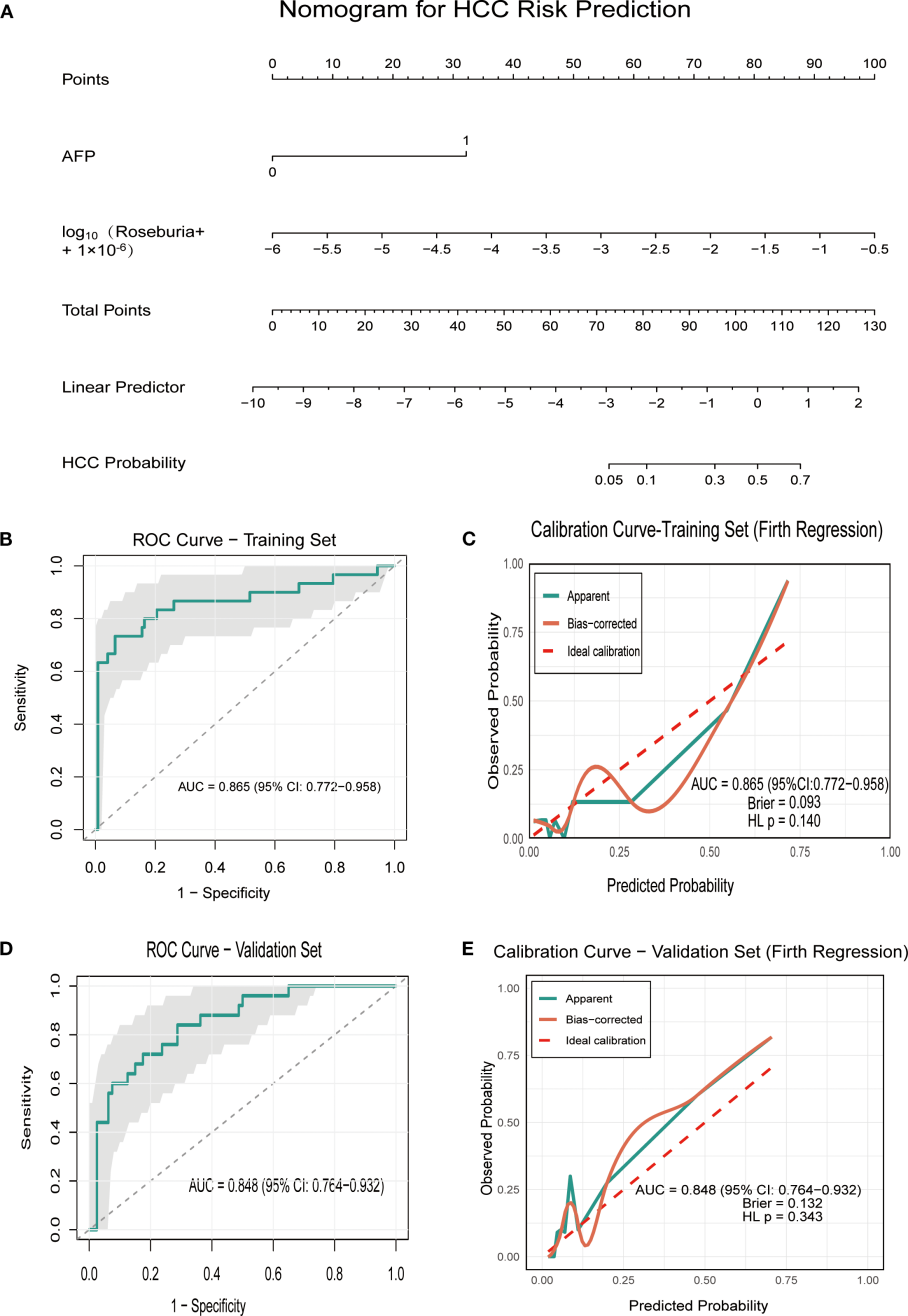
Performance of the PM-HCC predictive model. **(A)** Nomogram for predicting HCC risk (PM-HCC model) **(B)** The ROC curve of HCC predictive model (PM-HCC) in the training group; **(C)** Calibration curve of the PM-HCC model in the training group (Firth Regression); **(D)** The ROC curve of HCC predictive model (PM-HCC) in the validation group; **(E)** Calibration curve of the PM-HCC model in the validation group. ROC, receiver operating characteristic; PM-HCC, HCC predictive model.

To further investigate the significance of specific microbiota in distinguishing HCC, we performed univariate logistic regression analysis on four microbiota taxa independent of liver disease state and 10 clinical indicators. Among the 14 variables screened, six showed nominal significance (P < 0.05). Following false discovery rate (FDR) correction, three variables remained statistically significant (q < 0.05) and were subsequently included in multivariate analysis (**Supplementary Table S5**). The Firth regression analysis identified two independent predictors: elevated AFP (OR = 17.252, 95% CI: 6.622 – 50.319, p<0.001), and increased abundance of log_10_ *Roseburia* (OR = 4.695, 95% CI: 1.68 – 16.602, p =0.002). Multicollinearity diagnosis confirmed the stability of the final model, with a low correlation (Pearson’s r = 0.109) and variance inflation factors (VIF < 1.02) between the two predictors, indicating no substantial multicollinearity. Based on these findings, we preliminarily constructed an HCC prediction model (PM-HCC) using the following formula: Logit(P) = 0.273 + 2.848×AFP + 1.547×log_10_(*Roseburia* + 1×10^-6^). A clinical nomogram was developed to visualize the model for practical application (**Figure 5A**). ROC curve analysis demonstrated excellent discriminatory performance of the PM-HCC model, with an AUC of 0.865 (95% CI: 0.772–0.958) for HCC prediction (**Figure 5B**). The Hosmer–Lemeshow test (P = 0.140) and Brier score (0.093) confirmed good overall calibration and predictive accuracy (**Figure 5C**).

The temporal validation of the model was assessed in an independent validation cohort (n = 105, enrolled after January 1, 2021), including 20 cases of HC, 20 cases of CHB, 25 cases of HCC, and 40 cases of LC. The model maintained good discriminatory power for HCC, achieving an AUC of 0.848 (95% CI: 0.764–0.932) (**Figure 5D**), with no significant differences in baseline characteristics between the training and validation groups (**Supplementary Tables S6–S9**). The model showed no significant evidence of miscalibration, as indicated by a non-significant Hosmer–Lemeshow test (P = 0.343) and a Brier score of 0.1315. The calibration curve further demonstrated good agreement between predicted probabilities and observed outcomes across the risk spectrum (**Figure 5E**).

## 4 Discussion

The relationship between the intestinal microbiota and HCC has attracted increasing global attention, as dysbiosis of the gut microbiota is closely associated with HCC development (12). Several studies have explored microbial alterations in HCC; however, their findings have not been entirely consistent. Further investigations revealed substantial variation in intestinal microbiota across different liver disease states, even when the etiology was the same (HBV-related HCC) (9, 11). We noted that the proportions of HCC cases arising from CHB, CC, or DC varied among studies, and the clinical manifestations of DC-HCC are much more severe than those of CHB-HCC. Such heterogeneity may represent an important factor influencing the gut microbiota. In our study, the intestinal microbiota of CHB-HCC, CC-HCC, and DC-HCC groups were indeed different. With worsening of the underlying liver disease, potentially pathogenic bacteria increased, whereas beneficial bacteria decreased. A similar phenomenon was reported previously, with increased abundance of the opportunistic pathogen *Escherichia coli* in cirrhosis-related HCC, and enrichment of the symbiotic *Bacteroidaceae* in non-cirrhotic HCC (13). These findings suggest that the liver disease state is indeed a critical determinant of intestinal microbiota in HCC. Nevertheless, many earlier studies did not stratify patients by underlying liver disease, making it difficult to distinguish whether microbial alterations were driven by liver disease states or by HCC itself. To clarify this issue, we compared intestinal microbiota within the same liver disease state, with or without HCC. This approach enabled identification of microbial features that were attributable specifically to HCC. Our study identified four key bacterial genera associated with HCC across all liver disease states: *Roseburia*, *Veillonella*, *Megasphaera*, and *Paraprevotella*, all of which were enriched in HCC (**Figure 4D**).

*Veillonella*, a common Gram-negative anaerobe in the oral cavity and intestine, acts as an opportunistic pathogen producing lipopolysaccharide (LPS), thereby activating the LPS–TLR4 signaling pathway to promote epidermal growth factor expression, inhibit apoptosis, and facilitate HCC proliferation (7, 14). Clinical studies have shown that enrichment of *veillonella* in HBV-related HCC correlates with elevated circulating IL-6 and IL-10, reflecting a systemic inflammatory state (15). Differential enrichment of *Veillonella* has also been reported between HCC patients and healthy controls (16), and a large cohort analysis linked higher gut abundance of *Veillonella* to increased all-cause mortality, suggesting its potential as an independent predictor of poor prognosis in HCC (17). Beyond its pro-inflammatory effects, *Veillonella* ferments lactate into acetate, a key short-chain fatty acid (SCFA) (18). *Paraprevotella* is also an acetate-producing bacterium (19), and its abundance has been reported to be higher in early-stage HCC compared to cirrhosis (8). Acetate has emerged as a metabolite of particular interest in HCC. Zheng et al. (20) found that *Veillonella* abundance was associated with early recurrence of HBV-related HCC, accompanied by elevated acetate in tumor tissues. Mechanistically, acetate supplies acetyl-CoA via acyl-CoA synthetase short-chain family member 2 (ACSS2), thereby fueling tumor metabolism under stress (21, 22). Recent studies confirmed this tumor-promoting role, demonstrating that microbiota-derived acetate accelerated HCC progression by inducing hyper-O-GlcNAcylation–mediated metabolic reprogramming under high-fructose conditions (23). However, opposing effects of acetate have also been reported. Song et al. (24) demonstrated that acetate derived from *Bifidobacterium pseudolongum* suppressed fatty liver–associated HCC, while Ma et al. (25)found that acetate from *Bacteroides thetaiotaomicron* reshaped the immune microenvironment and inhibited tumor growth by modulating fatty acid metabolism and histone acetylation. Furthermore, acetate has been shown to suppress tumor-promoting immunity by inhibiting IL-17A production in hepatic type 3 innate lymphoid cells (ILC3s), thereby synergizing with PD-1 blockade to enhance antitumor responses (26). At the tumor-intrinsic level, acetate utilization via ACSS2 also exhibits heterogeneity: high ACSS2 expression was linked to greater acetate incorporation, reduced malignancy features, and improved prognosis, whereas low ACSS2 expression correlated with glycolytic and hypoxic phenotypes and poorer outcomes (27). Collectively, these findings underscore that acetate can both promote and suppress hepatocarcinogenesis in a context-dependent manner. Given this duality, future studies integrating metabolomics-based validation with mechanistic and longitudinal analyses will be essential to delineate the precise role of acetate in HCC.

*Megasphaera* produces key metabolites including SCFAs, amino acids, and vitamins, which are generally beneficial to host health (28). Nonetheless, increased *Megasphaera* abundance has been observed in gastric cancer (29), and Li et al. (30) reported enrichment of *Megasphaera* in HCC tumor tissues. More recently, both *Veillonella* and *Megasphaera* were reported to be increased in lung cancer and in elderly HCC patients (31, 32). Whether these taxa directly contribute to or promote HCC progression remains unclear. *Roseburia*, a Gram-positive anaerobe, is a well-recognized butyrate producer (33). In line with our findings, a recent study reported higher *Roseburia* abundance in HCC compared with cirrhosis (34). SCFAs exert diverse biological effects, among which butyrate serves as a major energy source for colonocytes, promotes epithelial proliferation and differentiation, and plays an essential role in intestinal immunity, inflammation, and barrier integrity (35). Butyrate has also been shown to suppress carcinogenesis by inhibiting tumor growth, inducing differentiation and apoptosis, and restraining uncontrolled proliferation (36). Our group previously demonstrated that microbiota-derived butyrate inhibited HCC growth by promoting natural killer cell infiltration in a CXCL11-dependent manner (37). Consistently, other studies showed that butyrate suppressed HCC progression by modulating intracellular Ca²^+^ homeostasis and enhanced the efficacy of sorafenib in preclinical models (38). It has also been reported to alleviate sorafenib resistance through microRNA-mediated mechanisms (39). Nevertheless, butyrate is not uniformly protective. Excessive SCFA concentrations can exacerbate colitis and promote tumorigenesis (40). Ma et al. (41) observed that *Roseburia* abundance was positively associated with lung cancer recurrence, with butyrate derived from *Roseburia* enhancing tumor cell proliferation, migration, and invasion. Similarly, Singh et al. (40) showed that long-term intake of fermentable fiber, which is converted to SCFAs by gut bacteria, promoted HCC development in dysbiotic mice. Okumura et al. (42) further demonstrated that intestinal bacteria from colorectal cancer patients contributed to tumorigenesis through butyrate secretion. These findings underscore that under dysbiotic or pathological conditions, butyrate may paradoxically promote tumor progression. Although *Roseburia* species are established butyrate producers, inter-strain variability in metabolic and immunomodulatory capacities may explain their divergent roles, with strain-dependent effects in HCC. *Agathobacter*, another butyrate producer (43), was reduced in CHB-HCC and CC-HCC patients in our study, consistent with previous reports (44), but showed a significant increase in DC-HCC. This contrasting pattern suggests that the role of *Agathobacter* in HCC is not an intrinsic characteristic but is instead shaped by the distinct pathophysiological context of the underlying liver disease. This suggests that while some butyrate producers may be protective, others may be enriched in HCC and contribute to local butyrate accumulation. Taken together, these results indicate that butyrate functions as a “double-edged sword”: it maintains intestinal and immune homeostasis and may exert anti-tumor effects under normal conditions, but under dysbiosis, excessive butyrate production may facilitate tumor progression.

Overall, the roles of microbiota-derived acetate and butyrate in HCC are complex, and current knowledge likely represents only a fraction of their effects. More comprehensive cellular, animal, and human studies, combined with bacterial culture experiments, are required to clarify their mechanisms. Importantly, alterations in intestinal microbiota, including shifts in butyrate-producing taxa, appear to be both a consequence and a driver of HCC across various liver disease states, and such microbial signatures may represent valuable diagnostic or prognostic indicators.

Previous studies have primarily constructed classification models (e.g., random forest) based on complex overall microbiome profiles to distinguish HCC patients from non-HCC individuals (8, 9). In contrast, our study developed and preliminarily validated an HCC prediction model that integrates the traditional serum biomarker alpha-fetoprotein with a single key microbiota biomarker, *Roseburia*. The model showed promising discriminative ability in both the training and independent validation cohorts, with AUC values of 0.865 and 0.848, respectively (**Supplementary Table S5**, **Figure 5**). Critically, it also demonstrated good calibration in the external validation set, as indicated by a non-significant Hosmer–Lemeshow test (P = 0.343) and a Brier score of 0.1315, suggesting that it may have potential clinical applicability. These findings suggest potential diagnostic utility, although the model should be regarded as preliminary and requires validation in larger, multicenter studies before clinical application can be considered.

However, the present study has several limitations. First, the sample size of the validation cohort was relatively small (n = 105), with particularly limited numbers in the high-risk subgroup, which may affect the stability of decision curve analysis. Second, the single-center design limits the generalizability of our findings. Third, although all groups were documented as consuming mixed diets (**Table 1**) and individuals who had used antibiotics, probiotics, or proton pump inhibitors within one month were excluded to minimize potential bias from diet and therapy, residual confounding cannot be completely ruled out. Finally, this study relied on 16S rRNA gene sequencing, which provides taxonomic resolution but only indirect inference of microbial function, whereas shotgun metagenomic sequencing would allow more comprehensive characterization of functional pathways. Future studies with larger, multicenter cohorts and metagenomic approaches, together with detailed recording of daily diet and therapeutic regimens, are warranted to more comprehensively assess the microbiota and its clinical implications. Furthermore, the analysis was constrained by the small subgroup of CHB-HCC patients, reflecting the low incidence of non-cirrhotic HBV-related HCC. For example, a U.S. study reported that only 30 (9.5%) of 317 HBV-related HCC patients did not have cirrhosis (45). Finally, the analysis was constrained by the use of relative abundance data for microbiota quantification, which lacks the precision of metagenomic sequencing.

Despite these limitations, our study demonstrates the significant value of integrating gut microbiota biomarkers to enhance the performance of HCC prediction models. The strong discriminatory power of this preliminary model and its acceptable overall calibration indicate that a strategy based on microbial markers is both feasible and effective. Future research should therefore prioritize validating and refining this model through prospective, multi-center studies with larger cohorts, specifically enriched for rare subgroups such as CHB-HCC to enhance generalizability and calibration. Additionally, efforts should focus on elucidating the biological mechanisms linking *Roseburia* to HCC, while employing more precise metagenomic sequencing techniques to improve microbial quantification. Future studies should also incorporate detailed dietary assessments and comprehensive documentation of therapeutic regimens to control for potential nutritional and treatment-related confounders, thereby better isolating the specific effects of the intestinal microbiota on HCC pathogenesis.

In conclusion, an imbalance of intestinal microbiota exists in patients with HBV-related HCC, which is influenced both by liver disease state and by HCC itself. The PM-HCC model, integrating microbiota and clinical indicators, showed potential clinical value for predicting HCC in patients with chronic HBV infection. Nevertheless, its application should be interpreted with caution, and external multicenter validation is warranted before clinical implementation.

## Data Availability

The data presented in this study are deposited in the NCBI Sequence Read Archive (SRA) repository, accession number PRJNA784025.
