# Supplementary material for: Featured intestinal microbiota associated with hepatocellular carcinoma in various liver disease states
Source: Front Immunol. 2025 Oct 6;16:1674838. doi: 10.3389/fimmu.2025.1674838 (PMC12536024; doi:10.3389/fimmu.2025.1674838)
Supplement: Supplementary file 1 [file DataSheet1.pdf]

Supplementary Methods

Calculation of sample size

According to the related literature, the mean values of cirrhosis dysbiosis ratio (CDR) in healthy group, compensated cirrhosis, decompensated cirrhosis and inpatients were 2.05, 0.89, 0.66 and 0.32 respectively(1), with a standard deviation of 1.5. The corresponding indicators were input into PASS (version 15) software, and calculate the total sample size of 100 cases, 25 cases in each group, according to the multi-group sample size calculation formula, take $\alpha=0.05$, $1-\beta=0.9$, each group 1:1:1:1 ratio. Due to the large heterogeneity of intestinal microbiota, it should be included as much as possible in the specified period of time.Two study cohorts were established based on the enrollment time, including a training group (including 60% of all subjects, n=152) and validation group (including 40% of all subjects, n=105), which consisted of 4 groups (HC, CHB, LC and HCC) in each cohort.

Gene extraction, PCR amplification, product purification, library construction and sequencing of fecal samples

The genomic DNA of fecal samples was extracted by Cetyltrimethylammonium bromide (CTAB) method. The Barcode-specific primers (the 16S V3-V4 region primer is 341F-806R: 341F-CCTAYGGGRBGCASCAG, 806R-GGACTACNNGGG TATCTAAT) and the Phusion ®High-Fidelity PCR Master Mix（New England Biolabs）with GC Buffer system were used for PCR amplification. Mix same volume of 1X loading buffer (contained SYB green) with PCR products and operate electrophoresis on 2% agarose gel for detection. Samples with bright main strip between 400-450bp were chosen for further experiments. PCR products was mixed in equidensity ratios. Then, mixture PCR products was purified with GeneJET Gel Extraction Kit(Thermo Scientific). Using Illumina TruSeq DNA PCR-Free Library Prepare Kit (Illumina, USA) to generate sequencing libraries, and assessing the library quality on the Qubit@ 2.0 Fluorometer (Thermo Scientific) and Agilent Bioanalyzer 2100 system. At last, the library was sequenced on an Illumina NovaSeq platform

References

1. Bajaj JS, Heuman DM, Hylemon PB, Sanyal AJ, White MB, Monteith P, et al. Altered Profile of Human Gut Microbiome Is Associated with Cirrhosis and Its Complications. *J Hepatol* (2014) 60(5):940-7. Epub 2014/01/01. doi: 10.1016/j.jhep.2013.12.019.
