# Supplementary material for: Featured intestinal microbiota associated with hepatocellular carcinoma in various liver disease states
Source: Front Immunol. 2025 Oct 6;16:1674838. doi: 10.3389/fimmu.2025.1674838 (PMC12536024; doi:10.3389/fimmu.2025.1674838)
Supplement: Supplementary file 2 [file DataSheet2.docx]

**Supplementary data**

**Featured Intestinal Microbiota Associated With Hepatocellular Carcinoma in various liver disease states**

**Running title: Microbiota and HCC**

Xiu Sun^1,2†^, Zhewen Zhou^1†^, Xin Chi ^1^ , Danying Cheng^1,3^, Yuanyuan Zhang^3,4^, Yifan Xu^1^, Yanxu Hao^1^ , Ying Duan^1,3^, Wei Li^1,3^, Yingying Zhao^1,3^, Shunai Liu^3,4^, Ming Han^3,4^, Xi Wang^3,4^, Song Yang^1,3^, Calvin Q. Pan^1,5*^ and Huichun Xing^1,4,6*^

1 Center of Liver Diseases Division 3, Beijing Ditan Hospital, Capital Medical University, Beijing, China

2 Shanxi Bethune Hospital, Shanxi Academy of Medical Sciences, Third Hospital of Shanxi Medical University, Tongji Shanxi Hospital, Taiyuan, China

3 National Center for Infectious Diseases, Beijing, China

4 Beijing Key Laboratory of Emerging Infectious Diseases, Institute of Infectious Disease, Beijing Ditan Hospital, Capital Medical University, Beijing, China

5 Division of Gastroenterology and Hepatology, NYU Langone Health, New York University School of Medicine, New York, NY, United States

6 Peking University Ditan Teaching Hospital, Beijing, China

**Supplementary Figures**

**Fig.S1.** Rarefaction curve and rank abundance curve of each sample.

**Fig.S2.** The relative abundance of intestinal microbiota in healthy control(HC), chronic hepatitis B (CHB), liver cirrhosis (LC) and hepatocellular carcinoma (HCC) at the phylum and genus level.

**Fig.S3.** The relative abundance of intestinal microbiota in CHB and CHB-HCC at the phylum and genus level.

**Fig.S4.** Differential taxa identified by LEfSe analysis in CHB and CHB-HCC. Selected taxa with a linear discriminant analysis score greater than 2.5 (LDA＞2.5).

**Fig.S5.** The relative abundance of intestinal microbiota in CC and CC-HCC at the phylum and genus level.

**Fig.S6.** Differential taxa identified by LEfSe analysis in CC and CC-HCC. Selected taxa with a linear discriminant analysis score greater than 2.5 (LDA＞2.5).

**Fig.S7.** The relative abundance of intestinal microbiota in DC and DC-HCCat the phylum and genus level.

**Fig.S8.** The α diversity (Chao1 index) of intestinal microbiota in DC-HCC was higher than that in DC.

**Fig.S9.** Differential taxa identified by LEfSe analysis in DC and DC-HCC. Selected taxa with a linear discriminant analysis score greater than 2.5 (LDA＞2.5).

**Fig.S1**

**
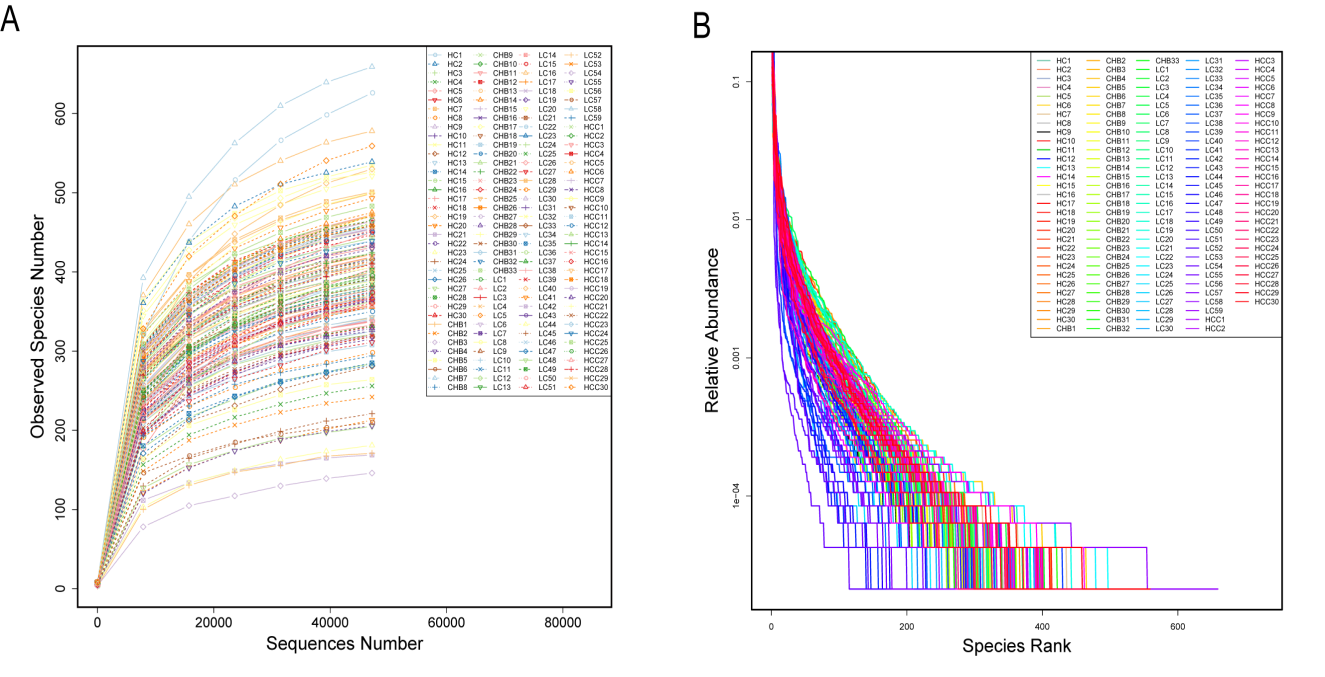
**

**Fig.S1. Rarefaction curve and rank abundance curve of each sample.** The curves tend to be flat, indicating that the sequencing depth was enough and the distribution of sample species was uniform. **(A)** Rarefaction curve. **(B)** Rank abundance curve.

**Fig.S2**

**
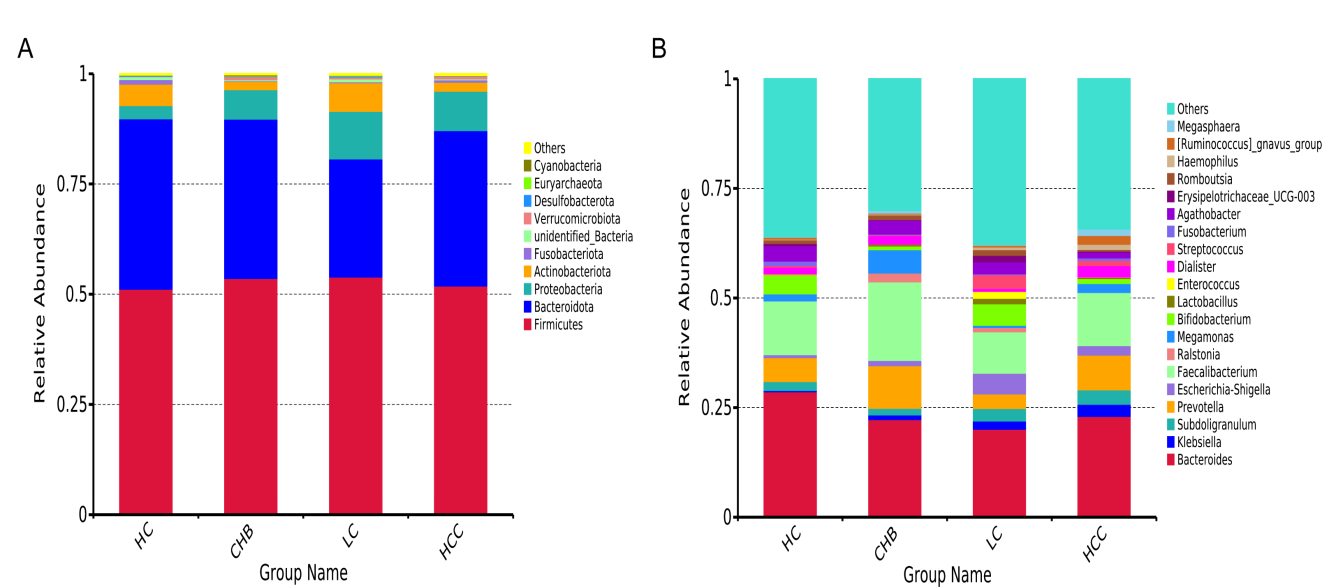
**

**Fig.S2. The relative abundance of intestinal microbiota in HC, CHB, LC and HCC at the phylum and genus level. (A)** The relative abundance of the top 10 phylum in four groups of intestinal microbiota. **(B)** The relative abundance of the top 20 genus in four groups of intestinal microbiota. HC, healthy controls; CHB, chronic hepatitis B; LC, liver cirrhosis; HCC, hepatocellular carcinoma.

**Fig.S3**


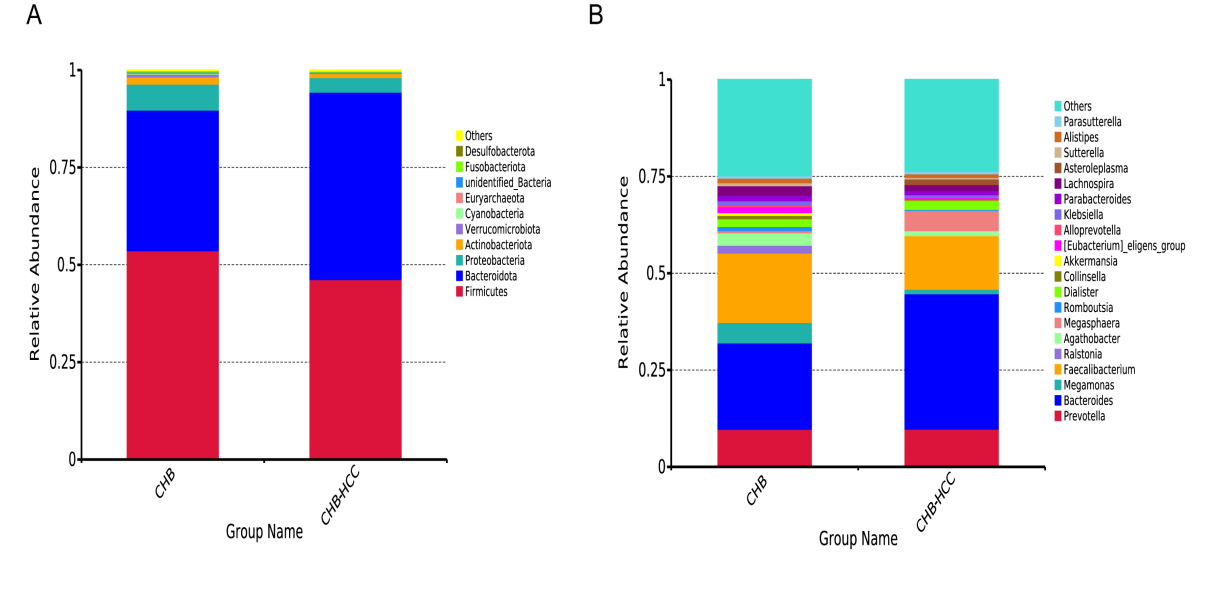


**Fig.S3. The relative abundance of intestinal microbiota in CHB and CHB-HCC at the phylum and genus level. (A)** The relative abundance of the top 10 phylum in two groups of intestinal microbiota. **(B)** The relative abundance of the top 20 genus in two groups of intestinal microbiota. CHB, chronic hepatitis B; CHB-HCC, HCC based on chronic hepatitis B.

**Fig.S4**


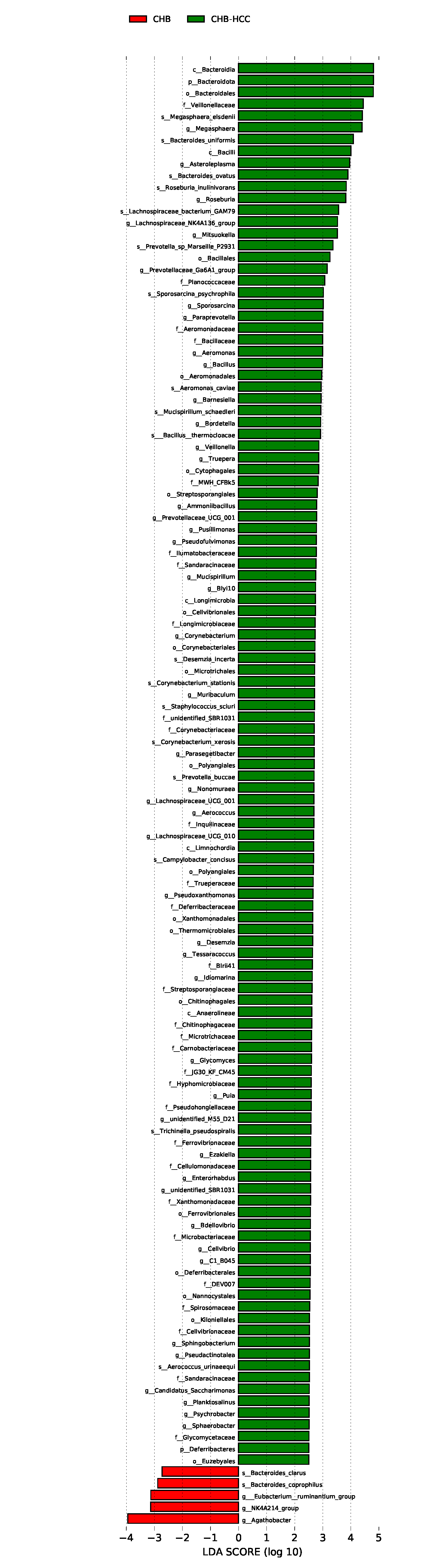


A

B


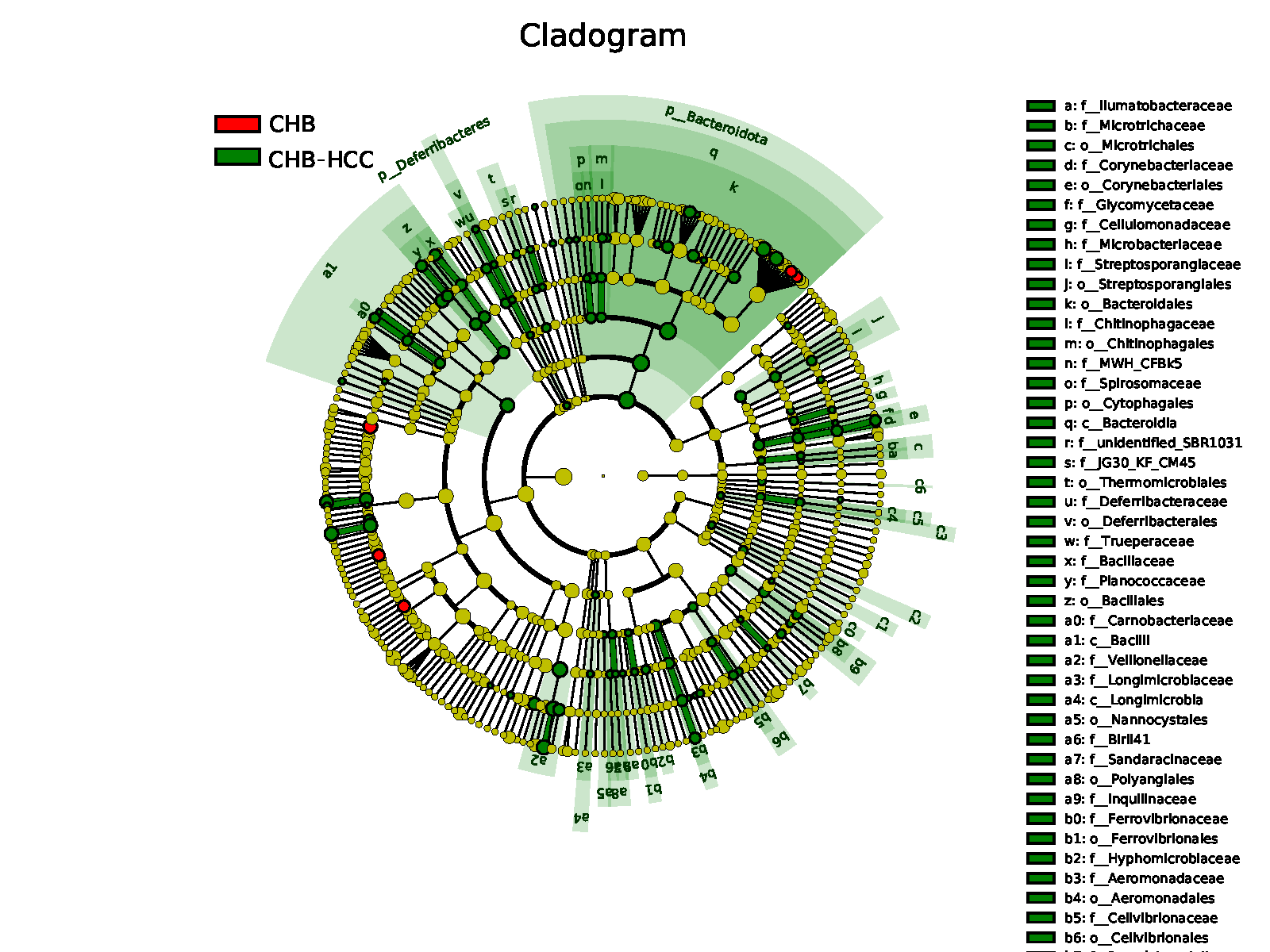


**Fig.S4. Differential taxa identified by LEfSe analysis in CHB and CHB-HCC. (A)** Histogram of the linear discriminant analysis scores (LDA＞2.5); The CHB-enriched taxa are indicated with a negative LDA score (red), and CHB-HCC-enriched taxa present a positive score (green). **(B)** Taxonomic cladogram obtained from LEfSe analysis of 16S sequences. The diameter of each circle was proportional to taxon abundance. CHB, chronic hepatitis B; CHB-HCC, HCC based on chronic hepatitis B.

**Fig.S5**

**
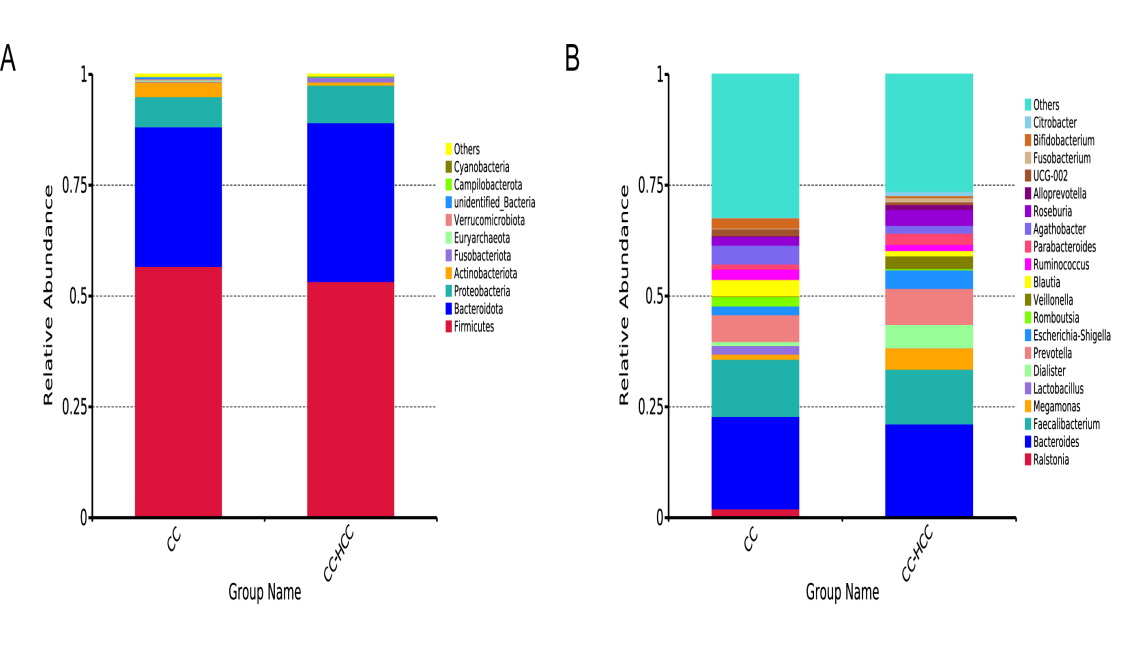
**

**Fig.S5. The relative abundance of intestinal microbiota in CC and CC-HCC at the phylum and genus level. (A)** The relative abundance of the top 10 phylum in two groups of intestinal microbiota. **(B)** The relative abundance of the top 20 genus in two groups of intestinal microbiota. CC, compensatory cirrhosis; CC-HCC, HCC based on compensatory cirrhosis.

**Fig.S6**

**
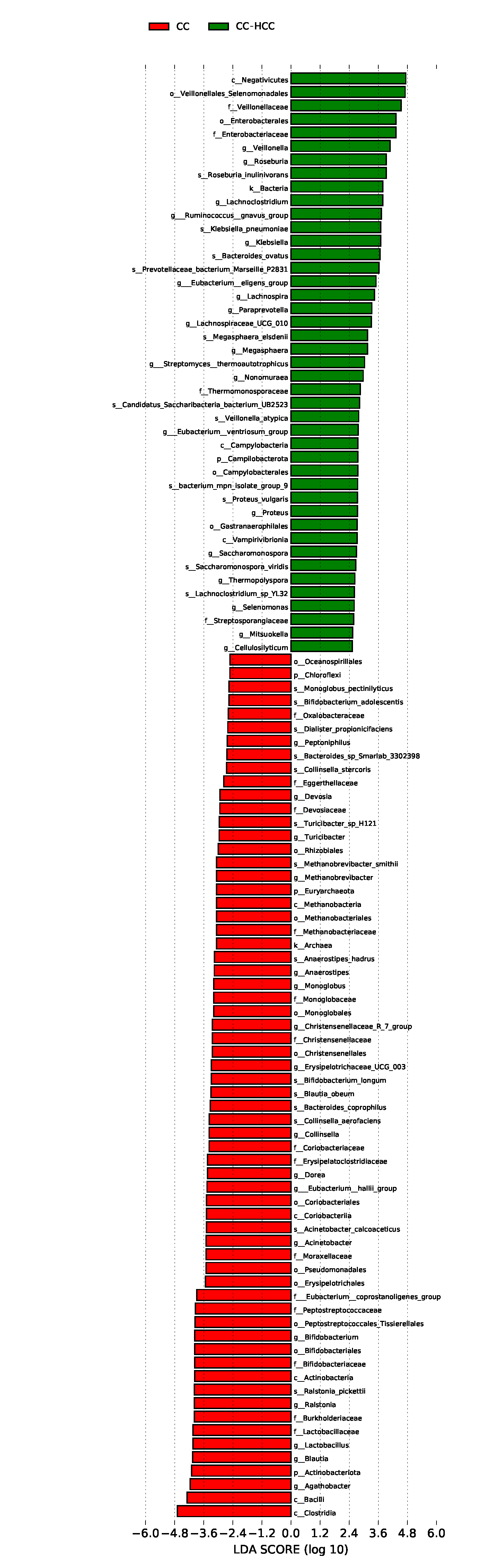
**

A

B


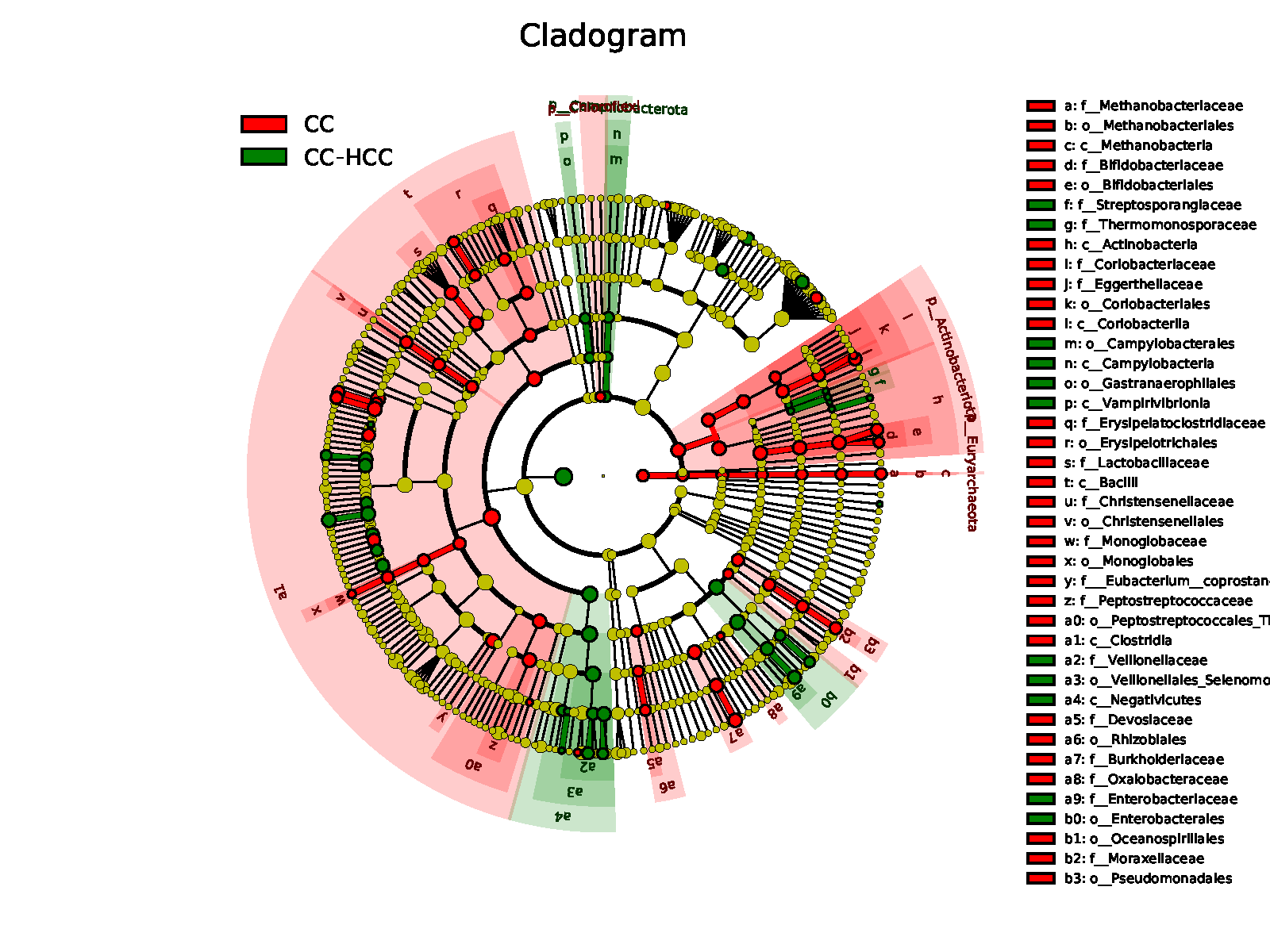


**Fig.S6. Differential taxa identified by LEfSe analysis in CC and CC-HCC. (A)** Histogram of the linear discriminant analysis scores (LDA＞2.5); The CC-enriched taxa are indicated with a negative LDA score (red), and CC-HCC-enriched taxa present a positive score (green). **(B)** Taxonomic cladogram obtained from LEfSe analysis of 16S sequences. The diameter of each circle was proportional to taxon abundance. CC, compensatory cirrhosis; CC-HCC, HCC based on compensatory cirrhosis.

**Fig.S7**

**
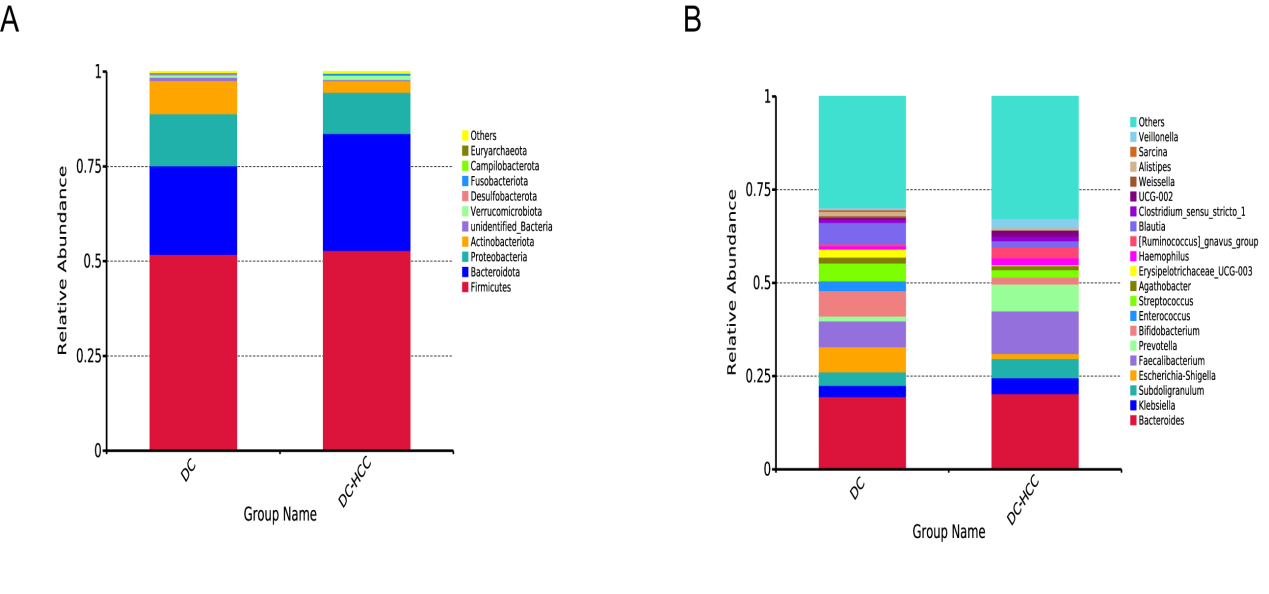
**

**Fig.S7. The relative abundance of intestinal microbiota in DC and DC-HCC at the phylum and genus level. (A)** The relative abundance of the top 10 phylum in two groups of intestinal microbiota. **(B)** The relative abundance of the top 20 genus in two groups of intestinal microbiota. DC, decompensated cirrhosis; DC-HCC, HCC based on decompensated cirrhosis.

**Fig.S8**


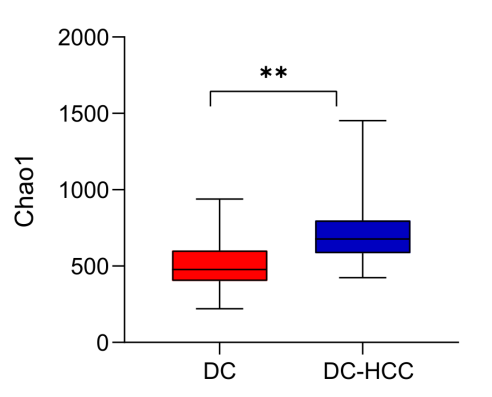


**Fig.S8. Comparison of α diversity (Chao1 index) of intestinal microbiota between DC and DC-HCC groups.** The Chao1 index of intestinal microbiota in DC-HCC was higher than that in DC. Significant differences (p < 0.05) are marked with an asterisk. DC, decompensated cirrhosis; DC-HCC, HCC based on decompensated cirrhosis.

**Fig.S9**

A


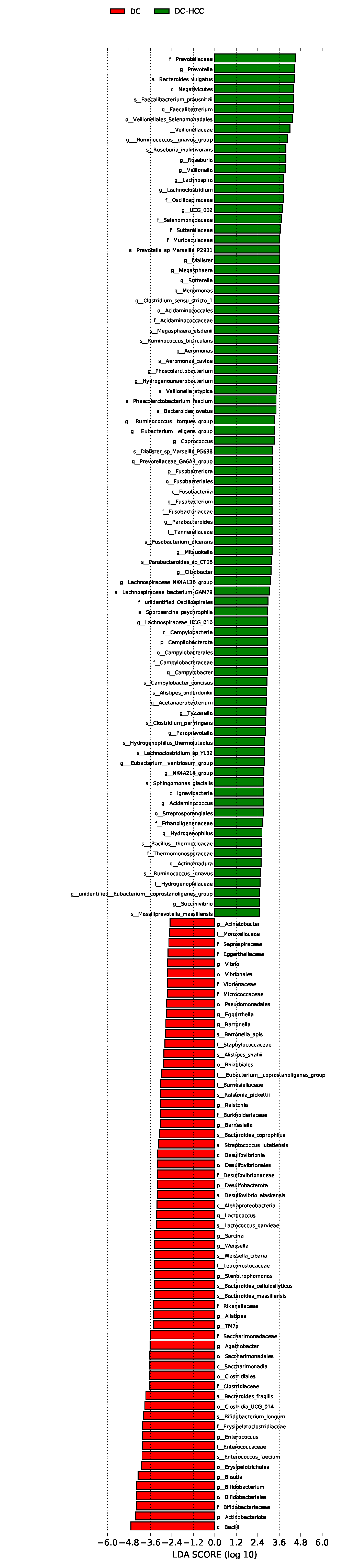


B


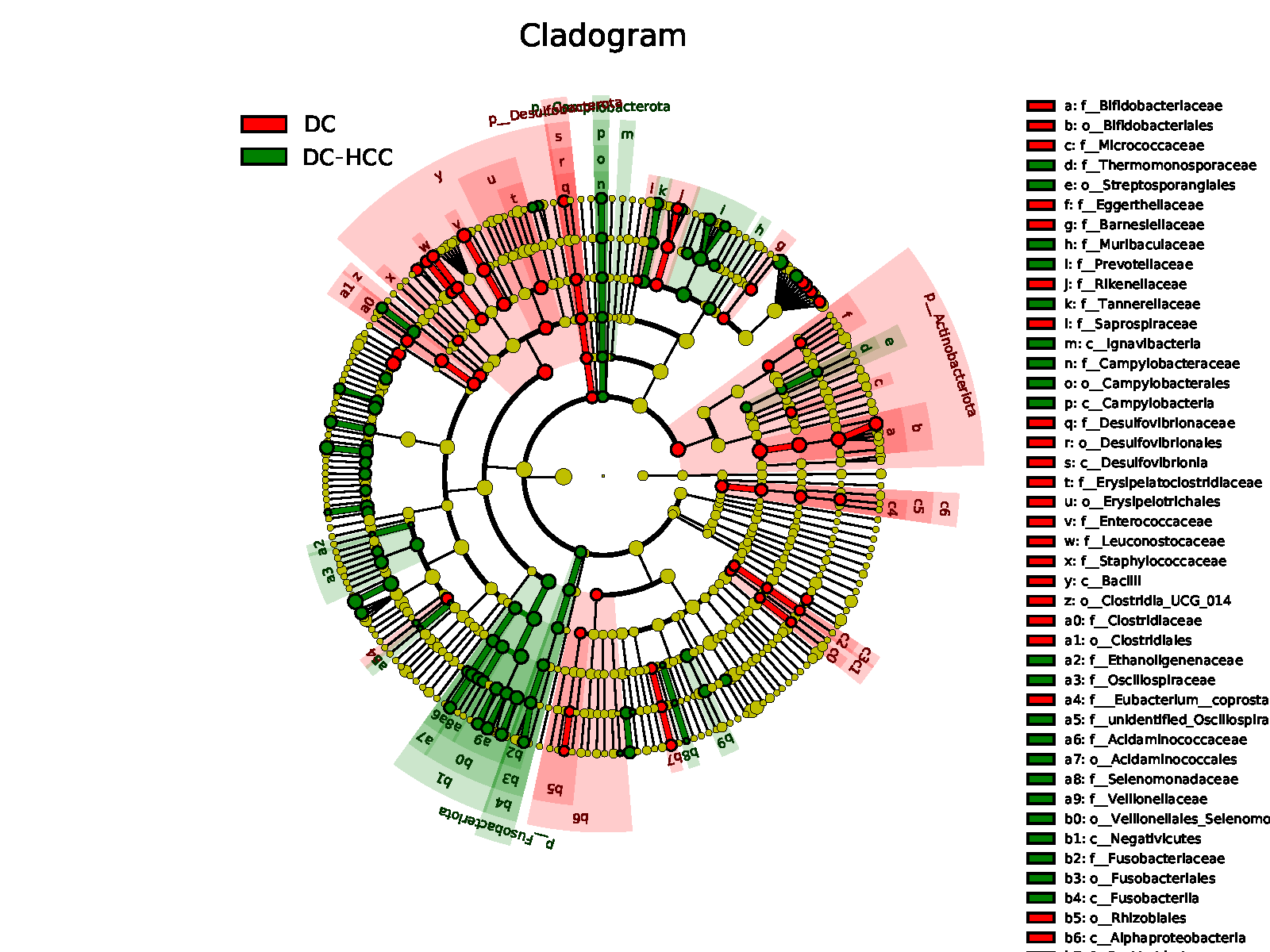


**Fig.S9. Differential taxa identified by LEfSe analysis in DC and DC-HCC. (A)** Histogram of the linear discriminant analysis scores (LDA＞2.5); The DC-enriched taxa are indicated with a negative LDA score (red), and DC-HCC-enriched taxa present a positive score (green). **(B)** Taxonomic cladogram obtained from LEfSe analysis of 16S sequences. The diameter of each circle was proportional to taxon abundance. DC, decompensated cirrhosis; DC-HCC, HCC based on decompensated cirrhosis.

**Supplementary Tables**

**Table S1.** Tumor size and number information of 30 patients with HCC

**Table S2**. Comparison of clinical and demographic features between CHB (n=33) and CHB-HCC ( n=5).

**Table S3.** Comparison of clinical and demographic features between CC (n=25) and CC-HCC(n=9).

**Table S4.** Comparison of clinical and demographic features between DC (n=34) and DC-HCC(n=16).

**Table S5.** Univariate and multivariate Univariate regression analysis of the risk of HCC.

**Table S6.** Comparison of clinical indicators of healthy controls (HC) between model group and verification group.

**Table S7.** Comparison of clinical indicators of CHB between model group and verification group.

**Table S8.** Comparison of clinical indicators of liver cirrhosis (LC) between model group and verification group.

**Table S9.** Comparison of clinical indicators of HCC between model group and verification group.

**Table S1：The size and number of tumors in HCC patients**

| Sample | Tumor size | Number of tumors |
| --- | --- | --- |
| HCC1 | 2cm | Single |
| HCC2 | Max.3.5cm | Multiple |
| HCC3 | 4.8cm | Single |
| HCC4 | 3.5cm | Single |
| HCC5 | 1.6cm | Single |
| HCC6 | 3.9cm | Single |
| HCC7 | 2.3cm | Single |
| HCC8 | Max.8cm | Multiple |
| HCC9 | Max.8cm | Multiple |
| HCC10 | 2.5cm | Single |
| HCC11 | Max.3cm | Multiple |
| HCC12 | Max.2cm | Multiple |
| HCC13 | 2cm | Single |
| HCC14 | Max.1.9cm | Multiple |
| HCC15 | 4.7cm | Single |
| HCC16 | Max.5.6cm | Multiple |
| HCC17 | 2cm | Single |
| HCC18 | 2.3cm | Single |
| HCC19 | Max.2.8cm | Multiple |
| HCC20 | 10cm | Single |
| HCC21 | 0.6cm | Single |
| HCC22 | 3.5cm | Single |
| HCC23 | 2.8cm | Single |
| HCC24 | 3.3cm | Single |
| HCC25 | 10cm | Single |
| HCC26 | 7.5cm | Single |
| HCC27 | Max.3cm | Multiple |
| HCC28 | 2cm | Single |
| HCC29 | Max.1cm | Multiple |
| HCC30 | 3cm | Single |

**Table S2: Clinical and demographic features of CHB and CHB-HCC**

| Characteristics | CHB  (n=33) | CHB-HCC  (n=5) |
| --- | --- | --- |
| Gender(F/M) | 10/23 | 1/4 |
| Age | 47.4±8.2 | 49.6±2.3 |
| BMI‾(x±S) | 24.1±1.8 | 24.6±1.4 |
| ALT(U/L) | 23.3 ±9.4 | 28.9±8.0 |
| AST(U/L) | 21.5±6.7 | 34.5±24.0 |
| ALB(g/L) | 46.8±3.7^a^ | 40.6±5.1^a^ |
| TBil(µmol/L) | 14.7±5.0 | 14.1±2.7 |
| Cr(µmol/L) | 71.2±11.6 | 69.9±11.7 |
| HbeAg | 6.6±13.7 | 1.8±2.0 |
| WBC(10^9^/L) | 5.7±1.0 | 5.7±1.3 |
| RBC(10^12^/L) | 5.0±0.4^a^ | 4.5±0.5^a^ |
| HGB(g/L) | 152.4±18.7 | 142.6±14.8 |
| PLT(10^9^/L) | 209.4±57.0 | 190.8±73.6 |
| INR | 1.06±0.10^a^ | 1.1±0.03^a^ |
| AFP(ng/ml) |  |  |
| ≤8.78* | 33（100%）^a^ | 1（20%）^a^ |
| ＞8.78 | 0（0%） | 4（80%） |

Note: Continuous variables were expressed as means ± standard deviation. Superscript letters indicated a significant difference (P < 0.05). *8.78 is the upper limit of the normal value of AFP. ALT, alanine aminotransferase; AST, aspartate aminotransferase; ALB, albumin; AFP, [alpha fetoprotein;](http://www.youdao.com/w/AFP(alpha%20fetoprotein)/#keyfrom=E2Ctranslation) BMI: body mass index; Cr, creatinine; TBil, total bilirubin; HBeAg: HBV e antigen; WBC, white blood cell; RBC, red blood cell; HGB, hemoglobin; PLT, blood platelet; INR, international normalized ratio; CHB,chronic hepatitis B; CHB-HCC, HCC based on chronic hepatitis B.

**Table S3: Clinical and demographic features of CC and CC-HCC**

| Characteristics | CC  (n=25) | CC-HCC  (n=9) |
| --- | --- | --- |
| Gender(F/M) | 10/15 | 3/6 |
| Age | 48.7±9.0 | 54.4±8.8 |
| BMI‾(x±S) | 24.1±1.6 | 24.2±2.4 |
| ALT(U/L) | 26.6±9.8 | 21.4±11.5 |
| AST(U/L) | 32.1±12.4 | 31.2±27.4 |
| ALB(g/L) | 42.3±5.0 | 39.4±2.4 |
| TBil(µmol/L) | 17.8±8.0 | 16.8±7.8 |
| Cr(µmol/L) | 65.0±12.0 | 71.2±11.4 |
| HbeAg | 3.3±7.2 | 0.5±0.4 |
| WBC(10^9^/L) | 4.5±1.4 | 4.5±1.8 |
| RBC(10^12^/L) | 4.7±0.5 | 4.4±0.4 |
| HGB(g/L) | 143.0±16.0 | 136.8±12.9 |
| PLT(10^9^/L) | 108.3±38.3 | 131.4±55.2 |
| INR | 1.14±0.16 | 1.12±0.09 |
| AFP(ng/ml) |  |  |
| ≤8.78 | 19(76%)^a^ | 2(22.2%)^a^ |
| ＞8.78 | 6(24%) | 7(77.8%) |

Note: Continuous variables were expressed as means ± standard deviation. Superscript letters indicated a significant difference (P < 0.05). ALT, alanine aminotransferase; AST, aspartate aminotransferase; ALB, albumin; AFP, [alpha fetoprotein;](http://www.youdao.com/w/AFP(alpha%20fetoprotein)/#keyfrom=E2Ctranslation) BMI: body mass index; Cr, creatinine;TBil, total bilirubin; HBeAg: HBV e antigen; WBC, white blood cell; RBC, red blood cell; HGB, hemoglobin; PLT, blood platelet; INR, international normalized ratio; CC, compensatory cirrhosis; CC-HCC, HCC based on compensatory cirrhosis.

**Table S4: Clinical and demographic features of DC and DC-HCC**

| Characteristics | DC  (n=34) | DC-HCC  (n=16) |
| --- | --- | --- |
| Gender(F/M) | 11/23 | 2/14 |
| Age | 52.4±9.0 | 52.9±9.8 |
| BMI‾(x±S) | 24.5±1.7 | 24.8±1.3 |
| ALT(U/L) | 50.1±78.7 | 39.5±29.9 |
| AST(U/L) | 58.2±76.2 | 56.6±30.1 |
| ALB(g/L) | 32.7±6.8 | 35.1±9.1 |
| TBil(µmol/L) | 42.7±37.3^a^ | 21.5±7.6^a^ |
| Cr(µmol/L) | 69.4±15.4 | 71.8±11.2 |
| HbeAg | 130.0±294.0 | 12.9±43.0 |
| WBC(10^9^/L) | 3.4±1.9 | 3.9±2.3 |
| RBC(10^12^/L) | 3.7±0.7 | 4.0±0.8 |
| HGB(g/L) | 113.9±18.8 | 122.5±20.9 |
| PLT(10^9^/L) | 61.9±30.8 | 99.1±79.6 |
| INR | 1.55±0.54 | 1.31±0.19 |
| AFP(ng/ml) |  |  |
| ≤ 8.78 | 21(61.8%)^a^ | 4(25%)^a^ |
| ＞8.78 | 13(38.2%) | 12(75%) |

Note: Continuous variables were expressed as means ± standard deviation. Superscript letters indicated a significant difference (P < 0.05). ALT, alanine aminotransferase; AST, aspartate aminotransferase; ALB, albumin; AFP,[alpha fetoprotein;](http://www.youdao.com/w/AFP(alpha%20fetoprotein)/#keyfrom=E2Ctranslation) BMI: body mass index; Cr, creatinine;TBil, total bilirubin; HBeAg: HBV e antigen; WBC, white blood cell; RBC, red blood cell; HGB, hemoglobin; PLT, blood platelet; INR, international normalized ratio. DC, decompensated cirrhosis; DC-HCC, HCC based on decompensated cirrhosis.

**Table S5:** **Univariate and multivariate Univariate regression analysis of the risk of HCC**

|  | Univariate analysis | | | Multivariate analysis(Firth regression) | | |  |
| --- | --- | --- | --- | --- | --- | --- | --- |
|  | OR (95% CI) | P | Q |  | OR (95% CI) | P | |
| **Bacteria** |  |  |  | **Bacteria** |  |  | |
| Veillonella | 4.271e^21^(1769.151~1.03e^40^) | 0.021 | 0.063 |  |  |  | |
| Roseburia | 7.252e^9^(747.591~7.034e^16^) | 0.006 | 0.042 | Log_10_Roseburia | 4.695 (1.68 - 16.602) | 0.002 | |
| Megasphaera | 3.912e^20^(374.991~4.08e^38^) | 0.025 | 0.063 |  |  |  | |
| Paraprevotella | 82.537（0~4.18e^12^） | 0.726 | 0.777 |  |  |  | |
| **Clinical indicators** |  |  |  | **Clinical indicators** |  |  | |
| Age | 1.046 (0.999 - 1.094) | 0.054 | 0.115 |  |  |  | |
| Gender | 2.177 (0.826 - 5.736) | 0.115 | 0.216 |  |  |  | |
| ALT | 1.001（0.991~1.010） | 0.902 | 0.902 |  |  |  | |
| AST | 1.005（0.997 ~1.014 ） | 0.205 | 0.334 |  |  |  | |
| ALB | 0.933（0.887 ~0.982 ） | 0.008 | 0.042 |  |  |  | |
| TBil | 0.987（0.959 ~ 1.015） | 0.347 | 0.473 |  |  |  | |
| WBC | 0.916（0.722 ~ 1.161） | 0.469 | 0.541 |  |  |  | |
| RBC | 0.518（0.304 ~0.883） | 0.016 | 0.059 |  |  |  | |
| PLT | 0.997（0.991 ~1.002） | 0.223 | 0.334 |  |  |  | |
| AFP | 17.812（6.702~47.336） | 0.000 | 0.000 | AFP | 17.252 (6.622 - 50.319) | < 0.0001 | |

**Abbreviations:** ALT, alanine aminotransferase; AST, aspartate aminotransferase; ALB, albumin; AFP, [alpha fetoprotein;](http://www.youdao.com/w/AFP(alpha%20fetoprotein)/#keyfrom=E2Ctranslation) TBil, total bilirubin; WBC, white blood cell; RBC, red blood cell; PLT, blood platelet.

**Table S6: Comparison of clinical indicators of healthy controls (HC) between model group and verification group.**

| Characteristics | HC1  (n=30) | HC2  (n=20) | p |
| --- | --- | --- | --- |
| Gender(F/M) | 12/18 | 3/17 | 0.059 |
| Age | 48.1±9.0 | 52.2±5.3 | 0.057 |
| BMI‾(x±S) | 23.37±2.9 | 24.8±1.3 | 0.766 |
| ALT(U/L) | 22.7 ±10.6 | 18.7±9.7 | 0.160 |
| AST(U/L) | 19.4±5.0 | 20.2±4.0 | 0.312 |
| ALB(g/L) | 45.3±2.8 | 46.6±2.3 | 0.071 |
| TBil(µmol/L) | 14.4±4.6 | 13.4±3.9 | 0.851 |
| Cr(µmol/L) | 66.4±13.0 | 74.6±14.0 | 0.064 |
| WBC(10^9^/L) | 5.2±0.9 | 5.5±1.1 | 0.184 |
| RBC(10^12^/L) | 4.8±0.3 | 4.8±0.4 | 0.500 |
| HGB(g/L) | 145.6±11.4 | 148.1±12.2 | 0.287 |
| PLT(10^9^/L) | 190.6±40.8 | 217.8±48.3 | 0.127 |
| AFP(ng/ml) | 3.0±1.3 | 3.0±1.1 | 0.968 |
| Dietary habit | Mix | Mix | - |

Note: Continuous variables were expressed as means ± standard deviation. ALT, alanine aminotransferase; AST, aspartate aminotransferase; ALB, albumin; AFP, [alpha fetoprotein;](http://www.youdao.com/w/AFP(alpha%20fetoprotein)/#keyfrom=E2Ctranslation) BMI: body mass index; Cr, creatinine; TBil, total bilirubin; WBC, white blood cell; RBC, red blood cell; HGB, hemoglobin; PLT, blood platelet.

**Table S7: Comparison of clinical indicators of CHB between model group and verification group.**

| Characteristics | CHB1  (n=33) | CHB 2  (n=20) | P |
| --- | --- | --- | --- |
| Gender(F/M) | 10/23 | 7/13 | 0.723 |
| Age | 47.4±8.2 | 43.8±8.2 | 0.123 |
| BMI‾(x±S) | 24.1±1.8 | 23.6±1.5 | 0.088 |
| ALT(U/L) | 23.3 ±9.4 | 22.2±8.6 | 0.607 |
| AST(U/L) | 21.5±6.7 | 22.4±5.7 | 0.457 |
| ALB(g/L) | 46.8±3.7 | 47.2±2.9 | 0.985 |
| TBil(µmol/L) | 14.7±5.0 | 14.3±8.6 | 0.244 |
| Cr(µmol/L) | 71.2±11.6 | 70.3±13.4 | 0.985 |
| WBC(10^9^/L) | 5.7±1.0 | 5.3±1.5 | 0.108 |
| RBC(10^12^/L) | 5.0±0.4 | 5.1±0.3 | 0.633 |
| HGB(g/L) | 152.4±18.7 | 150.4±14.6 | 0.557 |
| PLT(10^9^/L) | 209.4±57.0 | 199.3±74.8 | 0.804 |
| INR | 1.06±0.10 | 1.04±0.06 | 0.768 |
| AFP(ng/ml) | 2.84±1.38 | 3.94±3.87 | 0.086 |
| Log10HBVDNA(IU/ml) | 1.09±0.09 | 1.15±0.17 | 0.142 |
| Dietary habit | Mix | Mix | - |

Note: Continuous variables were expressed as means ± standard deviation. Superscript letters indicated a significant difference (P < 0.05). ALT, alanine aminotransferase; AST, aspartate aminotransferase; ALB, albumin; AFP,[alpha fetoprotein;](http://www.youdao.com/w/AFP(alpha%20fetoprotein)/#keyfrom=E2Ctranslation) BMI: body mass index; Cr,creatinine; TBil, total bilirubin; WBC, white blood cell; RBC, red blood cell; HGB, hemoglobin; PLT, blood platelet; INR, international normalized ratio; CHB, chronic hepatitis B.

**Table S8: Comparison of clinical indicators of LC between model group and verification group.**

| Characteristics | LC1  (n=59) | LC2  (n=40) | P |
| --- | --- | --- | --- |
| Gender(F/M) | 21/38 | 17/23 | 0.488 |
| Age | 50.9±9.1 | 47.8±12.7 | 0.130 |
| BMI‾(x±S) | 24.3±1.7 | 23.8±1.6 | 0.238 |
| ALT(U/L) | 40.1±60.8 | 58.2±91.5 | 0.806 |
| AST(U/L) | 47.1±59.5 | 64.3±94.2 | 0.613 |
| ALB(g/L) | 36.7±7.8 | 38.6±5.6 | 0.242 |
| TBil(µmol/L) | 32.1±31.2 | 28.9±31.7 | 0.576 |
| Cr(µmol/L) | 67.5±14.1 | 67.1±11.3 | 0.940 |
| WBC(10^9^/L) | 3.8±1.8 | 3.6±1.6 | 0.640 |
| RBC(10^12^/L) | 4.1±0.8 | 4.1±0.6 | 0.833 |
| HGB(g/L) | 126.2±22.8 | 123.7±23.0 | 0.540 |
| PLT(10^9^/L) | 81.6±30.8 | 92.3±55.7 | 0.716 |
| INR | 1.38±0.47 | 1.25±0.18 | 0.671 |
| AFP(ng/ml) | 30.1±81.8 | 24.0±95.5 | 0.06 |
| Log10HBVDNA(IU/ml) | 2.7±1.9 | 2.9±2.2 | 0.897 |
| Dietary habit | Mix | Mix | - |

Note: Continuous variables were expressed as means ± standard deviation. Superscript letters indicated a significant difference (P < 0.05). ALT, alanine aminotransferase; AST, aspartate aminotransferase; ALB, albumin; AFP, [alpha fetoprotein;](http://www.youdao.com/w/AFP(alpha%20fetoprotein)/#keyfrom=E2Ctranslation) BMI: body mass index; Cr, creatinine; TBil, total bilirubin; WBC, white blood cell; RBC, red blood cell; HGB, hemoglobin; PLT, blood platelet; INR,international normalized ratio; LC，liver cirrhosis.

**Table S9: Comparison of clinical indicators of HCC between model group and verification group.**

| Characteristics | HCC1  (n=30) | HCC2  (n=25) | P |
| --- | --- | --- | --- |
| Gender(F/M) | 6/24 | 6/19 | 0.721 |
| Age | 52.8±8.6 | 57.0±11.4 | 0.094 |
| BMI‾(x±S) | 24.6±1.7 | 24.2±1.0 | 0.105 |
| ALT(U/L) | 32.3±24.0 | 31.8±20.8 | 0.636 |
| AST(U/L) | 45.3±30.1 | 41.8±31.8 | 0.521 |
| ALB(g/L) | 37.3±7.3 | 36.3±6.0 | 0.654 |
| TBil(µmol/L) | 18.8±7.5 | 20.7±12.5 | 0.839 |
| Cr(µmol/L) | 71.3±10.9 | 69.5±14.8 | 0.488 |
| WBC(10^9^/L) | 4.4±2.0 | 4.9±1.9 | 0.250 |
| RBC(10^12^/L) | 4.2±0.7 | 4.2±0.9 | 0.748 |
| HGB(g/L) | 130.1±19.4 | 131.5±25.7 | 0.571 |
| PLT(10^9^/L) | 124.1±77.5 | 113.0±57.8 | 0.819 |
| INR | 1.22±0.18 | 1.25±0.12 | 0.137 |
| AFP(ng/ml) | 349.3±601.2 | 547.8±909.1 | 0.412 |
| Log10HBVDNA(IU/ml) | 2.21±1.51 | 2.7±1.87 | 0.328 |
| Dietary habit | Mix | Mix | - |

Note: Continuous variables were expressed as means ± standard deviation. Superscript letters indicated a significant difference (P < 0.05). ALT, alanine aminotransferase; AST, aspartate aminotransferase; ALB, albumin; AFP, [alpha fetoprotein;](http://www.youdao.com/w/AFP(alpha%20fetoprotein)/#keyfrom=E2Ctranslation) BMI: body mass index; Cr, creatinine;TBil, total bilirubin; WBC, white blood cell; RBC, red blood cell; HGB, hemoglobin; PLT, blood platelet; INR,international normalized ratio; HCC, hepatocellular carcinoma.
